# Acoustoelectronic nanotweezers enable dynamic and large-scale control of nanomaterials

**DOI:** 10.1038/s41467-021-24101-z

**Published:** 2021-06-22

**Authors:** Peiran Zhang, Joseph Rufo, Chuyi Chen, Jianping Xia, Zhenhua Tian, Liying Zhang, Nanjing Hao, Zhanwei Zhong, Yuyang Gu, Krishnendu Chakrabarty, Tony Jun Huang

**Affiliations:** 1 Department of Mechanical Engineering and Materials Science, Duke University, Durham, NC USA; 2 Department of Electrical and Computer Engineering, Duke University, Durham, NC USA

**Keywords:** Microfluidics, Nanoscience and technology, Acoustics

## Abstract

The ability to precisely manipulate nano-objects on a large scale can enable the fabrication of materials and devices with tunable optical, electromagnetic, and mechanical properties. However, the dynamic, parallel manipulation of nanoscale colloids and materials remains a significant challenge. Here, we demonstrate acoustoelectronic nanotweezers, which combine the precision and robustness afforded by electronic tweezers with versatility and large-field dynamic control granted by acoustic tweezing techniques, to enable the massively parallel manipulation of sub-100 nm objects with excellent versatility and controllability. Using this approach, we demonstrated the complex patterning of various nanoparticles (e.g., DNAs, exosomes, ~3 nm graphene flakes, ~6 nm quantum dots, ~3.5 nm proteins, and ~1.4 nm dextran), fabricated macroscopic materials with nano-textures, and performed high-resolution, single nanoparticle manipulation. Various nanomanipulation functions, including transportation, concentration, orientation, pattern-overlaying, and sorting, have also been achieved using a simple device configuration. Altogether, acoustoelectronic nanotweezers overcome existing limitations in nano-manipulation and hold great potential for a variety of applications in the fields of electronics, optics, condensed matter physics, metamaterials, and biomedicine.

## Abstract (web summary)

Precise and dynamic manipulation of nano-objects on a large scale has been challenging. Here, the authors introduce acoustoelectronic nanotweezers, combining precision of electronic tweezers with large-field dynamic control of acoustic tweezers, demonstrating complex patterning of sub-100 nm objects.

## Introduction

The direct patterning and assembly of nanomaterials on a large scale has long been recognized as an attractive approach for fabricating nano-textured materials with highly controllable and tunable material properties. In the 1980s, optical tweezers were developed as a leading choice for nanoscale manipulation due to their unrivaled spatial resolution^1^ and single-particle maneuverability^2–6^. However, high optical field intensities (100–1000 kW mm^−2^) may lead to adverse effects including undesired photothermal convection, overheating, and damage to biological specimens^7–9^. Alternatively, plasmonic-based techniques allow for the robust trapping of nanoparticles with low field intensities (0.1–1 kW mm^−2^) by confining the light energy near metallic nano-antennas^8,10–16^, yet these plasmonics-based strategies lack reconfigurability since their nanostructure layouts are fixed after fabrication. In contrast, optically induced electrokinetic nanotweezers do not rely on fixed plasmonic structures, but instead make use of light patterns and opto-electrically-responsive substrates to enable dynamic nanoparticle patterning and concentration^17–20^. By electrically labeling the particles and media using surfactants, opto-thermoelectric nanotweezers^21^ can achieve similar nanomanipulation capabilities using lower intensity (0.05–0.4 kW mm^−2^) lasers and associated thermoelectric gradients. Optoelectronic tweezers^22–25^, on the other hand, utilize virtual electrodes created by illuminating a photoconductive substrate with low intensity (0.001–1 kW mm^−2^) light patterns to dynamically manipulate microparticles. For the nanomanipulation scenario, this strategy has evolved by combining light-induced dielectrophoresis with localized convective vortices^26^ to achieve nanoscale trapping, where the latter are unfavorable for deterministic manipulation beyond conductive nanoparticles. Generally, with all the advances of current nanomanipulation technologies, it is still challenging to develop a label-free, dynamically controllable method that can provide sufficiently large forces to enable nanomanipulation without introducing considerable background disturbances, especially when attempting to manipulate nanoparticles over a large area.

Alternatively, acoustics^27–36^ can be a promising strategy for dynamic nanoscale manipulation due to its ultra-low field intensity (0.0001–0.1 W mm^−2^)^27,37^, precision^38,39^, biocompatibility^27,40^, and ability to perform dynamic control of particles over a large spatial region (on the order of mm to cm)^41–47^. These approaches typically rely on acoustic radiation forces to trap particles in acoustic pressure nodes or antinodes. However, such strategies are fundamentally constrained by acoustic streaming, an inherent phenomenon that arises from wave propagation^48–50^, which can counteract the acoustic radiation forces and prevent the stable trapping and patterning of nanoparticles^51–54^. Furthermore, the particle-driving acoustic radiation forces diminish quickly as the particle diameter scales down below 100 nm. Even when employing acoustically induced dielectrophoretic forces, nanoscale objects cannot be robustly manipulated beyond metallic nanoparticles (e.g., silver nanowires^55^), which experiences large electrical forces to overcome the effects of acoustic streaming and sustain their positions. Due to these fundamental constraints, the deterministic manipulation of nanoparticles smaller than 100 nm^56,57^ has not been demonstrated using acoustic tweezers.

Here, we demonstrate acoustoelectronic nanotweezers (AENT), which combine the advantages of electrics and acoustics, to enable massively parallel, dynamic manipulation of sub-100 nm particles. AENT employ the transient charges^58^ coupled with the local elastic deformations propagating on a piezoelectric material to produce large yet dynamically controllable actuation forces with minimal background noise (i.e., hydrodynamic interference) and maximal field intensities, and thus allow the trapping and patterning of nanoparticles below 10 nm and the creation of complex nanotextures on a macroscopic scale. Compared with previous acoustic tweezing technologies, a key innovation of our AENT technology is the minimization of the hydrodynamic interference that arises from acoustic streaming. In our configuration, out-of-plane vibrations and the associated acoustic streaming are minimized while maintaining the maximum allowable in-plane deformations, which creates strong electric fields for stable nanomanipulation with minimal background interference. Using AENT, we demonstrate spatiotemporal control of complex patterns of nanomaterials across a large wave-interfering region. By varying the tonal parameters (e.g., frequency, phase, time, and amplitude), diverse nanomanipulation functions, including transportation, concentration, orientation, pattern-overlaying, and sorting, have been achieved using a simple device configuration. AENT enable precise, versatile, and dynamic manipulation of nano-objects on a macroscopic scale, and can be applied to the bottom-up design and creation of nanotextures and materials with highly tunable properties.

## Results

### Generating acoustoelectronic fields with minimal hydrodynamic disturbances

**Fig. 1:**
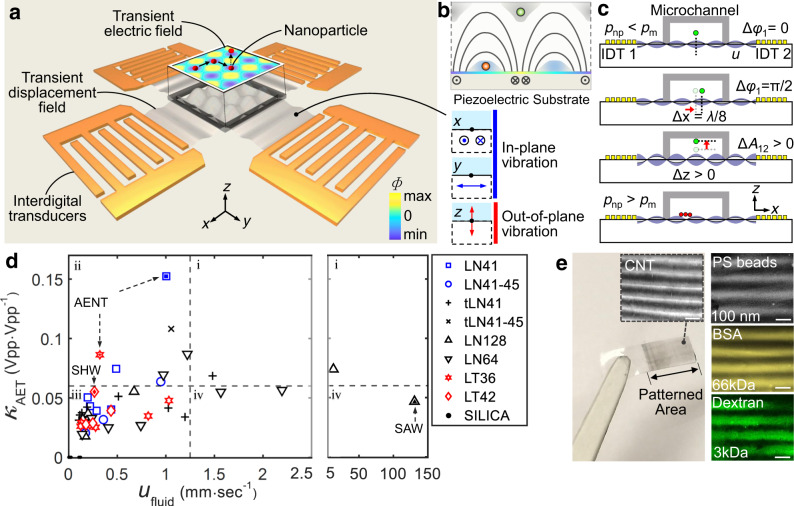
Working principles of acoustoelectronic nanotweezers (AENT). **a** Acoustoelectronic fields are generated via dynamic acoustic wave interactions. These acoustic waves have minimal out-of-plane vibrations and associated acoustic attenuation losses in a fluid. *F* is the surface electric potential. **b** Schematic side-view of the electric field distribution and trapping positions for particles with different polarizabilities relative to the medium (red sphere: high polarizability; green sphere: low polarizability). **c** Schematic mechanism of AENT on manipulating nanoparticles with lower (*p*_np_ < *p*_m_) or higher (*p*_np_ > *p*_m_) *p*olarizability than the medium in 3D space by tuning the phases and amplitudes of the acoustic waves. Δ*φ*_1_ indicates the phase variation of IDT_1_. Δ*A*_12_ indicates the amplitudes variation of IDT_1_ and IDT_2_. **d** Candidate excitation configurations based on nine potential single-crystal piezoelectric materials for AENT. *κ*_AET_ is the acoustoelectronic efficiency, which is defined as the ratio between the surface electric potential and the excitation voltage on the transducer in a standing wave mode. *u*_fluid_ is the acoustic streaming speed under consistent excitation amplitudes on different crystals. **e** Macroscopic materials with pre-designed nanotextures fabricated by AENT. Insets: microscopic images of PDMS films containing aligned carbon nanotubes and 100 nm PS beads, PEG hydrogels containing textured FITC-BSA proteins (66 kDa) and FITC-dextran (3 kDa). Scale bar: 60 μm.

As shown in Fig. 1a, interdigital transducers (IDT) fabricated on a piezoelectric substrate are used to generate local elastic deformations. Those deformations propagate as acoustic waves along the surface, interfere with each other and the boundaries, and finally establish electric fields with spatiotemporal periodicity from the coupled charges (i.e., acoustoelectronic fields). Note that the in-plane (i.e., *x*- and *y-*mode) displacements dominate the surface vibration to ensure strong acoustoelectronic fields for nanomanipulation, while the out-of-plane vibrations (i.e., *z*-mode) are minimized to remove the associated disturbances originating from acoustic streaming. Figure 1b depicts the schematic trapping positions for nanoparticles with different polarizabilities (green: low; red: high) with respect to the surrounding medium. Using AENT, tonal parameters such as frequencies, phases, and amplitudes can be encoded in the acoustoelectronic waves to enable various nanomanipulation functions with dynamic reconfigurability. For example, nanoparticles can be translated along horizontal directions (i.e., *x* and *y*) or vertical directions by tuning the phase (Fig. 1c, Δ*φ*_1_ = *π*/2) or amplitude (Fig. 1c, Δ*A*_12_ > 0). Theoretically, particles with lower (*p*_np_ < *p*_m_) or higher (*p*_np_ > *p*_m_) polarizability than the medium will be levitated in the fluid over the displacement antinodes or attracted to the substrate across the displacement nodes, respectively.

When implementing designs, the surface acoustoelectronic efficiency and acoustic streaming disturbances are the key factors that determine the performance of AENT. To be specific, higher acoustoelectronic efficiencies are desired on AENT to enable more efficient electrical manipulation, while lower acoustic streaming potentials can help reduce hydrodynamic disturbances and improve the stability of the manipulation capabilities. Here we selected nine different single-crystal piezoelectric materials that each have good potentials for generating in-plane vibrations for characterization in the frequency domain regarding these two factors (i.e., surface acoustoelectric efficiency and acoustic streaming). The surface acoustoelectric efficiency (*κ*_AET_) is defined as the ratio between the amplitude of surface electric potential (***F***) and the excitation amplitude in a continuous standing wave configuration, while the capabilities for generating acoustic streaming (*u*_fluid_) for different crystals are characterized by measuring the streaming speed in the fluid using particle image velocimetry in a continuous traveling wave regime. The scatter plot Fig. 1d summarizes the testing results on the potential working conditions of the nine potential piezoelectric materials filtered from the frequency-dependent data of surface acoustoelectronic efficiency and acoustic streaming speed. Both the positive signs of *κ*_AET_ and our simulation suggest that the *x*-mode displacement dominates the surface vibrations at the frequencies of interest. Note that *Y*-128 LiNbO_3_ is also tested due to its popularity in existing acoustic tweezers devices involving surface acoustic waves (solid black upper triangle)^59–61^, indicating the leaked acoustic energy and hydrodynamic disturbances are very high in the surface acoustic wave mode. As shown in Fig. 1d, among the four quadrants (i.e., i, ii, iii, iv), the excitation conditions of piezoelectric materials in quadrant ii have the highest *κ*_AET_ and lowest *u*_fluid_ for robust nanomanipulation. We found that *Y*-41 LiNbO_3_ and *Y*-36 LiTaO_3_ piezoelectric substrates have high acoustoelectronic efficiencies (e.g., *κ*_AET_ > 0.16 Vpp Vpp^−1^) and low acousto-hydrodynamic disturbances as compared with *Y*-128 LiNbO_3_ and *Y*-42 LiTaO_3_ substrates that have traditionally been used in acoustic tweezing devices^59–61^. Experimentally, since *Y*-41 LiNbO_3_ requires ~2-times lower excitation voltages than *Y*-36 LiTaO_3_ (e.g., for patterning 100 nm polystyrene beads), therefore it is employed as the main piezoelectric workhorse for AENT.

Using AENT with optimized substrates and excitation conditions, nanoscale particles and colloids can be patterned over a large area (e.g., centimeter scale). In particular, macroscopic materials with nanotextures can be fabricated with AENT by pattern transferring. As shown in Fig. 1e, patterns with aligned carbon nanotubes, 100 nm polystyrene beads, bovine serum albumin proteins (BSA, 66 kDa, ~3.5 nm), and dextran (3 kDa, ~1.4 nm) are created using AENT, which can subsequently be transferred to films of polydimethylsiloxane, hydrogel (e.g., GelMa, polyethylene glycol), and UV-epoxy upon polymerization. Furthermore, by utilizing the dynamic controllability of AENT and the spatiotemporal superposition of acoustic waves, those aligned carbon nanotubes can be actively interconnected together, thus creating anisotropic resistivities in the transferred patterns (Supplementary Fig. 1).

### Nanomanipulation with 1D acoustoelectronic fields

**Fig. 2:**
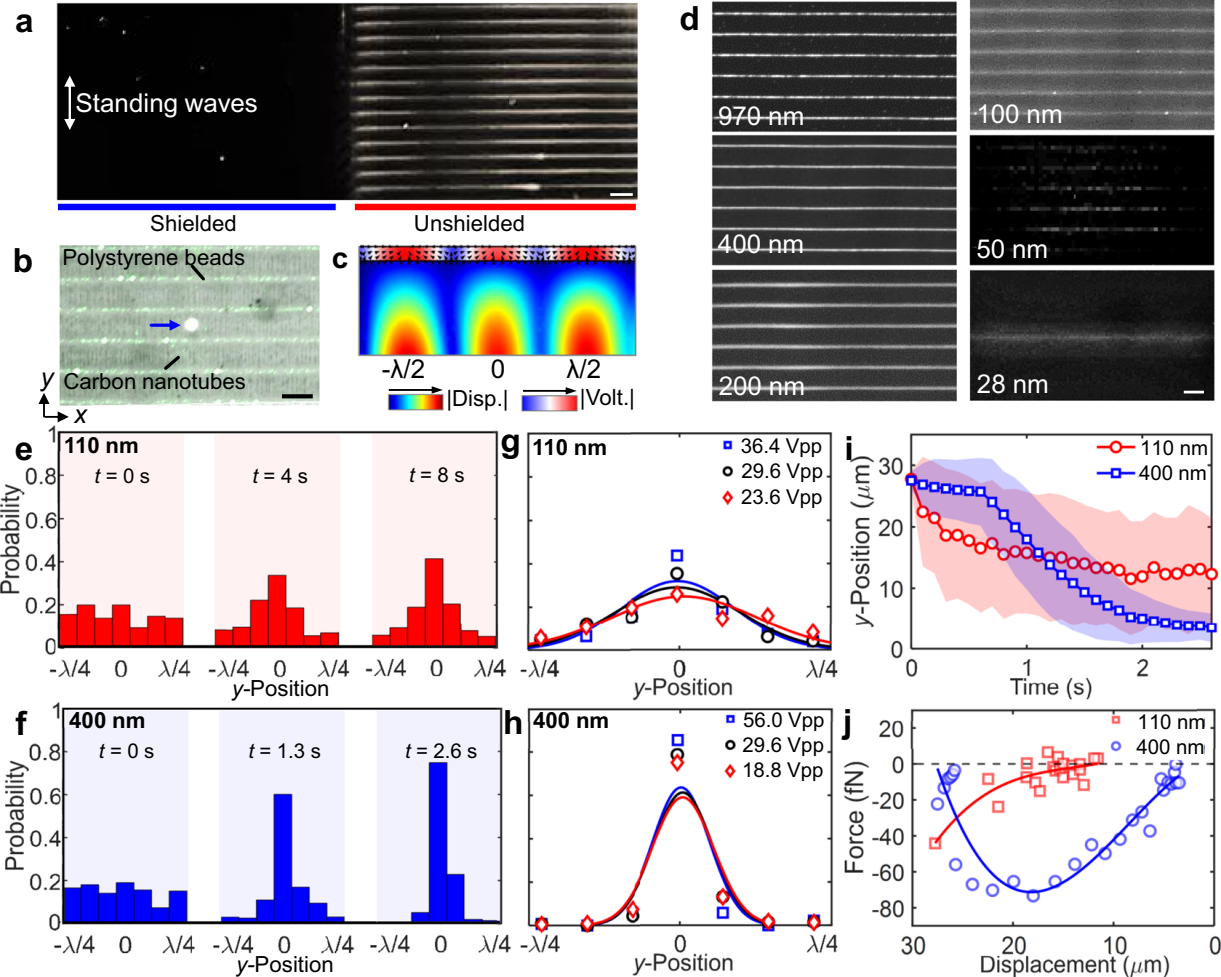
Nanoparticle manipulation with 1D standing waves. **a** Patterning of 100 nm diameter fluorescent polystyrene particles using 1D acoustoelectronic fields on a half-shielded piezoelectric substrate. **b** A stacked composite of images demonstrating the trapping positions of polystyrene nanoparticles (100 and 400 nm beads) and carbon nanotubes. The bright metal dot (blue arrow) indicates the geometric center between two mirrored IDTs with electric connection in *trans*-mode (Δ*φ*_1_ = π), of which the lateral position is aligned with a displacement node. **c** Simulation indicating the trapping position of 100 nm polystyrene beads in water. The arrow indicates the direction of the acoustoelectronic forces. Disp. substrate displacement, Volt. voltage in the water chamber. **d** 1D patterns of 28–970 nm polystyrene beads. **e**–**j** Nanoparticle image velocimetry analysis on 100 and 400 nm particles. **e**–**f** Time-elapsed distribution of single nanoparticles near the trapping nodes from −*λ*/4 to +*λ*/4. Normal fitting curves are used. **g**, **h** The influences of excitation amplitudes on the nanoparticle distribution. **i** The average trajectory of single nanoparticles under a 36.4 Vpp excitation amplitude. **j** The averaged distance-dependent actuation force on single nanoparticles. Scale bars: 60 μm.

To confirm that the working mechanism of AENT is due to acoustoelectronic effects, 100 nm polystyrene particles are tested in 1D standing waves over a region that is partially electrically shielded. As shown in Fig. 2a, no patterns or significant fluid motion have been observed over the electrically shielded region (Supplementary Movie 1), while clear linear patterns can be observed over the non-shielded area within 2 s of the acoustic waves being applied. This result indicates the dominance of acoustoelectronic effects over acoustic radiation forces despite the enhancement of acoustic contrast factors upon thermoviscous boundary effects^62^ for nanoscale particles. Figure 2b demonstrates the trapping positions of polystyrene particles (a mixture of 100 and 400 nm beads, *p*_np_ < *p*_m_) and carbon nanotubes (*p*_np_ > *p*_m_) inside a 25-μm-high chamber with a 1D standing acoustoelectronic waves. In this experiment, the differences in the surface conductance and electrical polarizability between the different nanoparticles determine their trapping positions (i.e., either along displacement nodes or antinodes)^63^. Such trapping positions can be predicted by the acoustoelectronic contrast or the real part of the Clausius–Mossotti factor^17,18^ (Eqs. (1)–(2)):

1 $${\rm{AEC}}={\rm{Re}}({\bf{C}}{\bf{M}})={\rm{Re}}(\frac{{{\boldsymbol{\varepsilon }}}_{{\bf{p}}}-{{\boldsymbol{\varepsilon }}}_{{\bf{m}}}}{{{\boldsymbol{\varepsilon }}}_{{\bf{p}}}+{{\boldsymbol{\varepsilon }}}_{{\bf{m}}}})$$

2 $${{\boldsymbol{\varepsilon }}}_{{\bf{m}}}={\varepsilon }_{\rm{{m}}}+{\rm{i}}\frac{{\sigma }_{\rm{m}}}{2\pi \it{f}};{{\boldsymbol{\varepsilon }}}_{{\bf{p}}}={\varepsilon }_{\rm{p}}+{\rm{i}}\frac{{\sigma }_{\rm{c}}}{2\pi \it{f}};{\sigma }_{\rm{c}}={\sigma }_{\rm{p}}+\frac{2{\sigma }_{\rm{s}}}{\it{r}}$$

where ${\sigma }_{\rm{{m}}}$, ${\sigma }_{\rm{{p}}}$, ${\varepsilon }_{\rm{{m}}}$, ${\varepsilon }_{\rm{{p}}}$, $f$, and $r$ are the conductivities of the medium and the particle, the dielectric constants of the medium and the particle, the frequency of the acoustic waves, and the radius of the particle, respectively. ${\sigma }_{\rm{{c}}}$ and ${\sigma }_{\rm{{s}}}$ are the composite conductivity and surface conductivity of the particle. On the one hand, a negative acoustoelectronic contrast factor indicates the lower polarizability of nanoparticle and as a result, the particle will be trapped over the displacement antinodes in the 1D standing wave field. On the other hand, a positive acoustoelectronic contrast factor indicates the higher polarizability of the nanoparticle, and the particle will be trapped over the displacement node in the 1D standing field. The time-averaged acoustoelectronic force $\left\langle {F}_{\rm{{AENT}}}\right\rangle$ acting on the nanoparticle can be calculated by^17,18^ Eq. (3):

3 $$\langle {F}_{\rm{{AENT}}}\rangle =2{\rm{\pi }}{\it{r}}^{3}{\varepsilon }_{\rm{m}}{\rm{Re}}({\bf{C}}{\bf{M}})\nabla {|{{\bf{E}}}_{{\rm{rms}}}|}^{2}$$

where ${{\bf{E}}}_{{\rm{rms}}}$ and the RMS value of electric field strength.

Experimentally, as shown in Fig. 2b, the carbon nanotubes (positive contrast) are patterned across the displacement nodes on the surface of the substrate and the 100 and 400 nm polystyrene beads (negative contrast) are aligned over the displacement antinodes near the ceiling in the 1D acoustoelectronic field. Such distributions match well with our simulated acoustoelectronic fields (Fig. 2c) with a calculated field strength maximum around |**Ex**| = 2.8 × 10^5^ V cm^−1^ and ∇|**Ex**_rms_|^2^ = 5.5 × 10^15^ V^2^/m^3^ along the *x*-axis upon 36.4 Vpp excitation. Furthermore, to remove concerns of cluster patterning, such 1D patterns can be resolved at the single-nano-particle level in experiments (Supplementary Movie 1). Interestingly, on substrates that are commonly used in acoustic tweezers, namely *Y*-128 LiNbO_3_, polystyrene microbeads are patterned over the displacement nodes of transverse waves due to the dominance of acoustic radiation forces. Figure 2d demonstrates the patterning of sub-micron and nanoscale fluorescent particles with sizes of 970, 400, 200, 100, 50, and 28 nm with 1D standing acoustoelectronic waves (Supplementary Movie 2), indicating the wide size range of maneuverable nanoparticles by AENT. Particularly, to pattern 28 nm beads, the working region of AENT is confined to one half-wavelength to increase the field intensity, thus allowing for the focusing of those nanoparticles into a single profile.

To characterize 1D nanomanipulation quantitatively, nanoparticle image velocimetry analysis is performed to analyze the particle distributions and trajectories under different experimental conditions. Figure 2e, f demonstrates the time-lapsed distribution of 110 and 400 nm polystyrene beads near the lateral trapping position within the half-wavelength. Note that the 400 nm particles can be patterned three times faster than the 110 nm beads. In addition, the 110 nm particles can only be stably patterned with excitation amplitudes within 23.6–36.4 Vpp (Fig. 2g); further increasing the excitation amplitude leads to a noticeable enhancement of the acoustic streaming that may disturb those nanopatterns. On the other hand, the 400 nm particles are insensitive to the excitation amplitude and form more rigid patterns (Fig. 2h). Additionally, the average trajectories of single 110 and 400 nm particles (i.e., 36.4 Vpp, >25 μm away from trap) are characterized. As shown in Fig. 2i, the single 400 nm particles can be shifted by an average of 0.73⋅*λ*/4 (i.e., 22 μm), while the single 100 nm particles can only be shifted by 0.5⋅*λ*/4 (i.e., 15 μm) with larger positional variances within 2 s. Based on the displacement-time relationship, the distance-dependent forces driving these averaged single-nanoparticle trajectories are calculated (Fig. 2j).

**Fig. 3:**
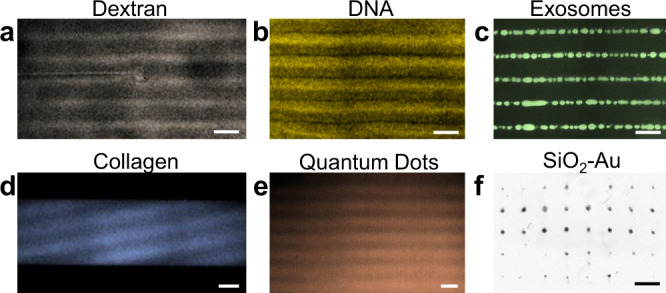
Manipulation of various nanomaterials and macromolecules via AENT. Patterning of **a** FITC-dextran (500 kMW), **b** stained DNA (3 kbp), **c** fluorescent urinary exosomes (30–150 nm), **d** FITC-collagen (type-I, 300 nm in length and 1.6 nm in diameter), **e** quantum dots (~6 nm CdSeS/ZnS), and **f** ~100 nm SiO_2_-Au particles. The backgrounds of the images have been removed and pseudo-colors are added to enhance visibility for these nanoscale samples. Scale bars: 60 μm; Collagen: 150 μm.

Various nanoscale materials, vesicles, or macro-biomolecules can be manipulated upon wave superposition using the acoustoelectronic effect. As shown in Fig. 3, different nano-analytes, including FITC-Dextran (500 kDa), DNA (3 kbp), FITC exosomes (30–150 nm), FITC-collagen (type-I, 300 nm in length and 1.6 nm in diameter), CdSeS-ZnS quantum dots (6 nm), and SiO_2_-Au nanobeads (~100 nm) as surface-enhanced-Raman-scattering (SERS) probes, can be patterned with 1D acoustoelectronic fields. Some inorganic nanomaterials, like graphene flakes (30–40 nm), zinc-oxide nanofibers (~1 μm long, ~100 nm in diameter) and seeds (~30 nm), can also be patterned into similar textures as carbon nanotubes. Interestingly, under high excitation amplitudes, abnormal patterns of FITC exosomes and 100 nm beads (Fig. 3c and Supplementary Movie 3) are observed, which can potentially be attributed to coacervation or disruption of the electric double layer of the colloidal particles. The pattern of exosomes under low amplitude excitation is shown in Supplementary Fig. 2. For patterning exceedingly small particles like quantum dots, the combined effects of dielectrophoresis and electrokinetic flows may both contribute to the particle trapping similar to Wong’s research in 2009^64^. All patterns of these nanoparticles (except graphene and Au beads) will dissolve once the AENT is turned off. To move a step further beyond 1D nanopatterns, SERS probes are tested and concentrated into a dot array configuration to achieve highly-sensitive Raman spectroscopy (Supplementary Fig. 3, excitation <20 Vpp) which can be considered as a simple demonstration of the utility of AENT in biosensing applications.

### Dynamic electric field control via acoustic wave superposition

**Fig. 4:**
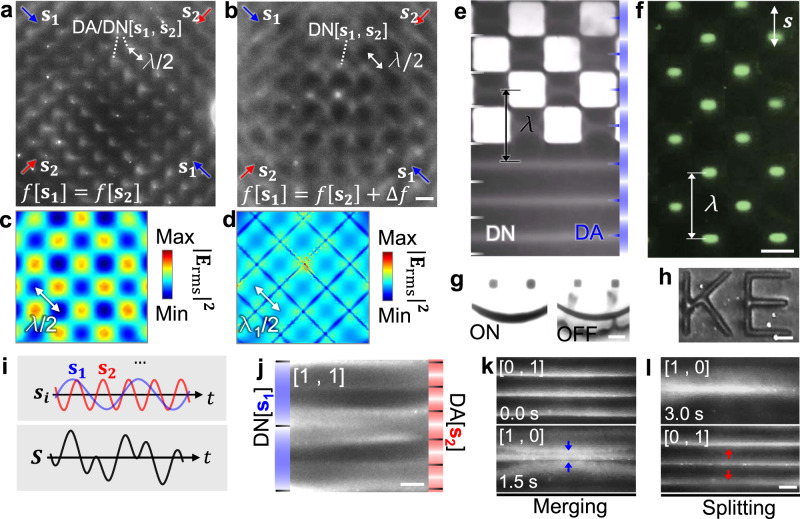
Dynamic electric field control via wave superposition. **a** Creating a 2D dot array of 100 nm polystyrene beads using orthogonal standing waves with identical frequencies (*f*[**s**_1_] = *f*[**s**_2_]). **b** The dot array pattern transforms to network with a mesh-like electric field distribution when *f*[**s**_1_] ≠ *f*[**s**_2_]. Numerically simulated magnitude of time-averaged electric field strength (${\left|{{\bf{E}}}_{{\rm{rms}}}\right|}^{{\boldsymbol{2}}}$) for **c** *f*[**s**_1_] = *f*[**s**_2_] and **d** *f*[**s**_1_] ≠ *f*[**s**_2_]. **e**–**h** Forming complex 2D nanopatterns with 1D waves. Checkered patterns of **e** carbon nanotubes and **f** 100 nm polystyrene beads with selectively shielded checkered areas. Forming arbitrary 2D patterns of (**g**) carbon nanotubes and (**h**) 100 nm polystyrene beads using phase-sweeping acoustoelectronic waves and selective shielding. The carbon nanotubes can be released once the excitat**i**on is off. **i** Harmonic standing waves (**s**_**i**_) with different tonal parameters are super-positioned to construct a complex electric field (**S**). When superimposing two harmonic waves (**s**_1_ and **s**_2_), the amplitudes are denoted a**s** a vector *Am*(**s**_1_, **s**_2_) = a[m, n]. **j** Composite patterns of 100 nm polystyrene beads on both displacement nodes (DN) and antinodes (DA) when [m, n] is equal to [1, 1] (*f* (**s**_**1**_) = 15.37 MHz, *f* (**s**_**2**_) = 38.44 MHz). **k** Merging and **l** splitting of linear patterns of 100 nm beads using spatiotemporal control of harmonic acoustic waves. The labels “**s**” with arrow indicate the propagation directions of the standing waves. Scale bars: 60 μm. **a** and **b**, **c** and **d**, **k** and **l** share the same scale bars, respectively.

Building upon the field-controllability of acoustoelectronic waves, AENT can be re-configured on-demand via wave superposition to achieve dynamic nanomanipulation. Specifically, tonal waves with varied parameters such as frequency, amplitude, phase, position, and time can be tuned to synthetize complex acoustoelectronic fields. For example, when two standing acoustoelectronic waves (*f*[**s**_1_] = *f*[**s**_2_]) are superimposed in 2D space orthogonally, 2D dot lattices of acoustoelectric fields and the associated 2D patterns of 100 nm polystyrene particles are created (Fig. 4a). In this scenario, the trapping positions of the beads used here are aligned to both displacement antinodes and nodes in the 2D-wave AENT configuration. When the frequencies are not identical (*f*[**s**_1_] ≠ *f*[**s**_2_]), the dot lattices transform to a network-like pattern^59^ (Fig. 4b). Such pattern transitions can be verified by simulating trapping potential distributions (i.e., ${\left|{{\bf{E}}}_{{\rm{rms}}}\right|}^{2}$, Fig. 4c, d). Furthermore, complex acoustoelectronic fields can also be created using 1D standing waves by electrically modifying the surface of the piezoelectric substrate. As shown in Fig. 4e, the 2D pattern of carbon nanotubes can be created using 1D waves on a selectively shielded region. Due to the different contrasts of the carbon nanotubes and polystyrene nanobeads, the 100 nm polystyrene particles are trapped over the center of the unshielded region due to the lower electric field strength and thus form a periodic array of concentrated dots (Fig. 4f). Such a concept can be adopted to create arbitrary nanopatterns (Fig. 4g, h) with phase-sweeping 1D waves, which could potentially be useful for fabricating nano-textured metamaterials with complex features. Beyond spatial modulation, temporal modulation of pulsed signals can also be applied to selectively create local patterns following a time-of-flight tweezing configuration (Supplementary Movie 4).

Harmonic standing waves (**s**_**i**_) with varied tonal parameters can be super-positioned on the same plane to form more sophisticated acoustoelectronic fields (**S**) (Fig. 4i). For example, two harmonic waves (**s**_**1**_ and **s**_**2**_) with identical parameters except for their frequencies can be superimposed together, and their amplitudes of the complex electric field can be denoted as a vector, namely **Am**(**s**_**1**_, **s**_**2**_) = a[m, n]. As shown in Fig. 4j, 100 nm polystyrene particles form composite nanopatterns under the composite field **Am**(**s**_**1**_, **s**_**2**_) = a[1, 1]. Note that the 100 nm particles used in **s**_1_ (*f*[**s**_1_] = 15.37 MHz) will be trapped along the displacement nodes (DN[**s**_1_]) due to its larger polarizability than the medium for *f*[**s**_1_], and the displacement antinodes (DA[**s**_2_]) in the standing wave **s**_2_ (*f*[**s**_2_] = 38.44 MHz) due to its smaller polarizability than the medium for *f*[**s**_2_] (Fig. 4j). Furthermore, the harmonic merging (Fig. 4k) and splitting (Fig. 4l) of linear patterns of 100 nm beads are realized by sequentially tuning the amplitude vectors **Am**(**s**_**1**_, **s**_**2**_) following [0, 1] to [1, 0] and [1, 0] to [0, 1], respectively (Supplementary Movie 5). With these demonstrated functionalities, the AENT enables highly sophisticated acoustoelectronic fields on a 2D surface via the spatiotemporal superposition of acoustic waves.

### Versatile nanomanipulation functions with AENT

**Fig. 5:**
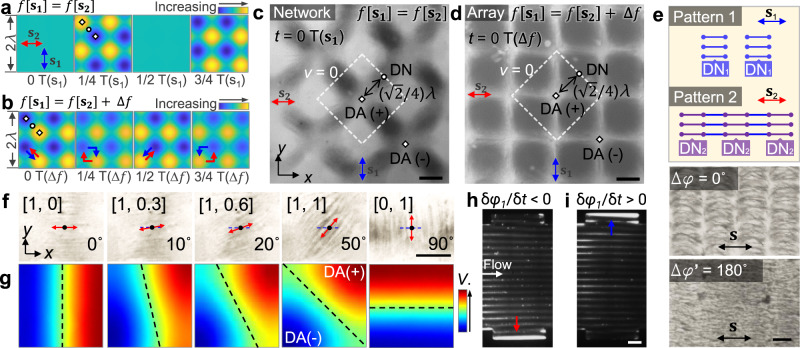
Various nanomanipulation functions (e.g., pattern transformation, orientation control, and enrichment) via AENT. **a**–**d** Pattern transformation of carbon nanotubes with 2D waves. Analytical, time-lapsed electric potential distribution for **a** scenario *f*(**s**_1_) = *f*(**s**_2_) at time 0, ¼, ½, and ¾ T(**s**_1_), and **b** scenario *f*(**s**_1_) ≠ *f*(**s**_2_) around time 0, ¼, ½, and ¾ T(Δ*f*). The diamond and the circular symbols indicate the displacement antinodes (DA) and displacement nodes (DN) in a transient electric field, respectively. The red and blue arrows in indicate the transition directions of DA(+) and DA(−), respectively. The carbon nanotube pattern transforms to form (**c**) networks or (**d**) a square-shaped array as a result of the two aforementioned excitation configurations. The white dashed lines indicate the contour of zero-potential (*v* = 0) for the transient electric field (*t* = 0 T). Label “T” represent the time period of the frequency components of *f*[**s**_1_] or Δ*f*. **e** Schematic and experimental images of creating interconnected graphene sheets with uniform orientation by overlaying different 1D patterns with phase variances. **f** Experimental demonstration and **g** simulations of the orientation control of nanomaterials (i.e., carbon nanotubes) by tuning the 2D amplitude distribution. **h**, **i** Enrichment of 100 nm polystyrene beads in continuous flow with boundary confinement using phase-sweeping acoustoelectronic waves. The blue and red arrows indicate the phase-shifting directions of standing waves. The pattern of nanoparticles continuously shifts (**h**) downward (*δφ*_1_/*δt* < 0) or (**i**) upward (*δφ*_1_/*δt* > 0) in flow and the nanoparticles finally become trapped in the groove on the corresponding side wall. The labels “**s**” with bi-directional arrows indicates the direction of standing waves. Scale bars: **c**–**f**: 30 μm; **h**, **i**: 120 μm.

Building upon the field-controlling methods described in previous section, AENT allows dynamic control of dielectric or metallic nanoparticles and can potentially be applied to a variety of applications involving nanoscale texturing, concentration, separation, and sorting. As shown in Fig. 5a, d, the 2D patterns of carbon nanotubes can be instantly transitioned between meshes and dot lattices by tuning the frequencies of the 2D orthogonal acoustoelectronic waves (**s**_1_, **s**_2_). Although the analytical transient voltage distributions are similar to the scenario of Fig. 3a, b (i.e., 2D patterns of polystyrene nanobeads), the local trapping positions (e.g., nodes, antinodes) are reversed due to the high polarizability of carbon nanotubes. To be specific, for *f*[**s**_1_] = *f*[**s**_2_] (Fig. 5a, c), the carbon nanotubes are aligned and patterned toward the displacement antinodes with opposite transient charges (white diamonds) across the displacement nodes (white circles). For the case of *f*[**s**_1_] = *f*[**s**_2_] + Δ*f* (Fig. 5b, Δ*f* is small), beside the main temporal periodicity near *f*[**s**_1_] and *f*[**s**_2_], the generated transient electric field also has another temporal periodicity of Δ*f* in the long term, leading to a dot array pattern (Fig. 5d and Supplementary Movie 6). Note that the red and blue arrows in Fig. 5b indicate the periodical shifting direction of the virtual electrodes with opposite transient charges (i.e., DA(+) and DA(−)) around time 0, 1/4, 1/2, 3/4 T(∆*f*), where T(∆*f*) = |1/(*f*[**s**_1_] − *f*[**s**_2_])|. As shown in Fig. 5d, the carbon nanotubes form a square-shaped array on the displacement nodes with the displacement antinodes coincident to the four corners of the square units and some interconnected carbon nanotubes aligned to the four edges of the square units.

Besides pattern transformation, the created nanopatterns can be overlaid to create more complex features. As shown in Fig. 5e, 1D graphene patterns can be overlaid to create desired nano-connectivity. Firstly, the graphene nanoflakes (30–40 nm, 5–7 atomic layers thick) are patterned by 1D AENT with high excitation amplitude (Pattern 1). Such high excitation amplitude ensures Pattern 1 firmly adheres to the substrate and will not be disturbed by subsequent acoustoelectronic fields or background flows. Then, more graphene solution is injected to the microfluidic chamber for creating Pattern 2, which also adheres to the substrate but with a position shift of *λ*/4, creating a thin, interconnected sheet of graphene nanoflakes with 1D connectivity. Here we only show a simple demonstration of pattern overlaying; however, there is a large parametric space for creating useful patterns or networks by pattern-overlaying and simultaneously tuning the tonal parameters.

For elongated nanomaterials, their orientations can be actively controlled by changing the amplitude ratios between the two orthogonally superimposed standing waves (i.e., **Am**[**s**_1_, **s**_2_] = [*A*_1_, *A*_2_]). As shown in Fig. 5f, the carbon nanotubes can be rotated with various amplitude vectors of [1, 0], [1, 0.3], [1, 0.6], [1, 1], and [0, 1] to different horizontal angles of 0, 10, 20, 50, and 90 degrees, respectively (Supplementary Movie 6). This rotation mechanism can be illustrated by the simulated transient electric potential (Fig. 5g): in the process of increasing *A*_2_, the zero-potential boundary between the virtual electrodes at two displacement antinodes (DA^+^ and DA^−^) rotates upon different amplitude combinations, changing the alignment directions of electric fields and carbon nanotubes.

The concentration of nanoparticles in continuous flow is important for sample isolation and enrichment for both diagnostics and therapeutics. Figure 5h, i demonstrates the enrichment of 100 nm polystyrene particles in flow by the synergies between continuously phase-shifting and channel boundaries on the side wall. Note that in this scenario, the orientation of the trapping nodes and the phase-shifting directions are parallel and perpendicular to the direction of flow, respectively. As the phase shifts from upward to downward (*δφ*_1_/*δt* < 0, Fig. 5h), the nanoparticles are continuously translated from right to left, and finally move along the channel wall following the flow until becoming trapped in the groove on the lower wall. Conversely (*δφ*_1_/*δt* > 0, Fig. 5i), the nanoparticles will be continuously enriched inside the groove on the upper side of channel wall.

### Nanomanipulation with single-particle maneuverability

**Fig. 6:**
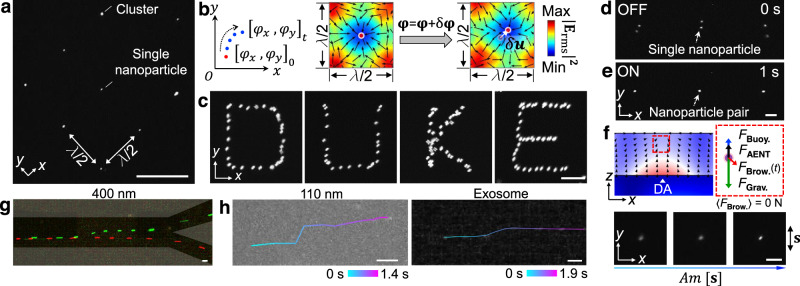
AENT enables nanomanipulation with single-particle precision. **a** 2D lattices containing single 400 nm polystyrene beads in several trapping wells. **b** Principle of 2D nanomanipulation by tuning the phases of the orthogonal standing waves. **c** Stacked fluorescence images showing a 400 nm polystyrene particle can be translated along complex paths as letters “D”, “U”, “K”, and “E”. **d**, **e** Reversible pairing of single 400 nm beads using 2D electric fields. **f** Schematic force analysis and levitation of a single 400 nm bead as the amplitude of the standing wave increases. *F*_AENT_ acoustoelectronic force, *F*_Buoy._ buoyant force, *F*_Grav._ gravitational force, *F*_Brow._ Brownian forces, DA displacement antinode. The label “**s**” with bi-directional arrows indicates the direction of standing waves. **g**, **h** Deflection of single nanoparticles in continuous flow using narrow acoustoelectronic wave beams. **g** Deflecting a 400 nm particle in a bifurcated channel. A single 400 nm particle flows to the lower outlet by-default (AENT off, red pseudo-color) or can be deflected to the upper channel when AENT is on (green pseudo-color). Gray shadings: PDMS wall. **h** Deflection of single 110 nm polystyrene bead, and single exosome particle in continuous flow. The time-elapse trajectories are plotted over the composite image, where the color scales indicate the elapsed time. Time intervals: 100 ms. Scale bars: **a**: 60 μm; **c**–**f**: 15 μm; **g**, **h**: 20 μm.

Due to the significant reduction in acoustic streaming, AENT allows for single-nanoparticle maneuverability. As a proof-of-concept demonstration, 400 nm fluorescent polystyrene particles, which can be stably tracked without surface adsorption, are used to explore single-nanoparticle maneuverability. As shown in Fig. 6a and Supplementary Movie 7, 400 nm particles can be patterned into a dot array with 2D AENT, and a portion of the trapping wells contain single particles. By dynamically tuning the phases of the 2D waves (i.e., ${\varphi }_{\rm{{x}}}$, ${\varphi }_{\rm{{y}}}$), the particles can be translated in the *x* and *y* directions with displacement $\delta {\bf{u}} = {\sum }_{0}^{t}(\lambda /4{\rm{\pi }})\delta {{\boldsymbol{\varphi }}}_{{\rm{t}}}$, where $\delta {{\boldsymbol{\varphi }}}_{\rm{t}}=[{\delta \varphi }_{\rm{{x}}},{\delta \varphi }_{\rm{{y}}}]_{t}$ (Fig. 6b). Based on this mechanism, a particle can be translated along complex routes in a programmable manner, forming the letters “D”, “U”, “K”, and “E” (Fig. 6c). Building upon this feature, parallel single-nanoparticle pairing becomes feasible to enable investigations in various fields including mechano-transduction, virology, and extracellular vesicle analysis with AENT. As shown in Fig. 6d, e, reversible single-particle pairing is realized by switching the 2D waves on and off repetitively (Supplementary Movie 7). Beyond horizontal control, the levitation of single nanoparticles is achieved by tuning the amplitudes of the standing acoustoelectronic waves to increase *F*_*AENT*_ (Fig. 6f). Note that although AENT can scale down to the single-nanoparticle level, the manipulation is not selective, since other particles in the wave-interfering region will also move when manipulating the particle of interest using the current device configuration. From our perspective, potential pathways for achieving selective single-nanoparticle manipulation may involve fine wave-front or time-of-flight control using holographic transducer arrays.

Narrow beams of acoustoelectronic waves can be used to selectively actuate single nanoparticles in a nano-sorter configuration. As shown in Fig. 6g, h, a single 400 nm particle, a single 110 nm particle, and a single exosome are deflected using standing acoustoelectronic beams, reaching deflection distances of 29 μm (Fig. 6g), 16 μm (Fig. 6h, 110 nm), and 11 μm (Fig. 6h, exosomes), respectively. Interestingly, on the *Y*-41 LiNbO_3_ substrate, those standing acoustoelectronic waves can be effectively confined into narrow beams (~1.67*λ*) by reducing the aperture of the IDTs; however, the focused IDT designs, which are typically used in traditional surface acoustic wave devices, are unable to reduce the beam width. AENT allows for robust nanomanipulation down to single-particle precision via the spatiotemporal super-positioning of acoustoelectronic waves.

## Discussion

In this article, we presented AENT as a multifunctional platform for the dynamic manipulation of nanomaterials on a large scale. By utilizing the transient electric fields coupled to acoustic waves, our method is able to manipulate sub-100 nm particles (e.g., DNAs, exosomes, ~30 nm graphene flakes, ~6 nm quantum dots, ~3.5 nm proteins, and ~1.4 nm dextran) in a massively parallel and dynamic manner. AENT inherit the advantages of acoustic manipulation, including dynamic reconfigurability, large field of control, and the ability to superimpose or modulate waves to create complex field distributions in a vast parametric space (e.g., *f*, *φ*, *A*, *t*, wave position, and acoustic/electric boundaries), as well as the advantages of electric manipulation, including simple device fabrication, precision, and convenient integration with other downstream platforms.

Like most nanotweezing techniques employing electrical effects, AENT should not operate in fluids with high ionic concentration due to issues like ionic shielding, Joule heating, and electrolysis of solvent. This observation suggests that non-ionic solutes (e.g., sucrose) are needed to maintain osmotic pressure for vesicles, limiting AENT’s potential to operate in native biofluids. However, unlike micro-scale living samples, most nanoscale materials and colloids (e.g., proteins, graphene) are not as sensitive as cells to the shock of buffer change. In this regard, the impact of buffer change can be minimized with properly controlled medium compositions for AENT.

By achieving precise, dynamic control over nanoparticles across a large spatial area, AENT overcomes a major limitation of existing nanomanipulation technologies. Specifically, in order to manipulate particles at the nanoscale, large actuation forces are required to enable precise control. However, as the magnitude of the actuation force increases, undesired thermal and hydrodynamic disturbances are typically introduced to the system, destabilizing the position of particles and thus preventing large-scale control. Through the combination of re-configurable acoustic waves and coupled electric fields, and simultaneous minimization of acoustic streaming, AENT achieves large actuation forces (fN–pN) on nanoparticles without introducing significant hydrodynamic disturbances. Various functions, including pattern translation, rotation, transformation, interconnection, particle pairing, levitation, concentration, and sorting, have been demonstrated based on the dynamic, re-configurable nature of the acoustoelectronic fields. Furthermore, the macroscopic patterns formed can be transferred to different materials or overlaid into complex patterns with desired interconnectivity (e.g., graphene sheets with 1D interconnectivity). We believe that AENT holds great potential as a next-generation nanomanipulation technology that will lead to applications across many disciplines such as nanofabrication, electronics, molecular physics, chemistry, metamaterials, and biomedicine.

## Methods

### Reagents and materials

The 3-inch single-side-polished *Y*-41 LiNbO_3_ wafers (and all other wafers) are purchased from Precision Micro-Optics Inc., MA, USA. The 4-inch single-side-polished silicon wafers are purchased from University Wafer Inc., MA, USA. The photoresist SPR-3012 is purchased from Kayaku Advanced Materials Inc., JP. SU-8 25 negative resist is purchased from MicroChem Inc., USA. The 28, 50, 100, 400 nm, and 2 μm fluorescence polystyrene beads are purchased from Magsphere Inc., CA, USA. The 200, 400, and 970 nm fluorescence beads are purchased from Bangs Laboratories Inc., IN, USA. We ordered customized 100 nm polystyrene particles with three-fold higher concentration of fluorescence dyes for nanoparticle image velocimetry from MagSphere Inc., CA, USA (mean diameter: 110 nm). The carbon nanotube water solution (multi-wall carbon nanotubes, 3 wt%, OD: 20–30 nm, length: 30 μm) is purchased from US Research Nanomaterials Inc., TX, USA. The PDMS Sylgard 184 is purchased from Ellsworth Adhesives Inc., WI, USA. The hard PDMS (PP2-RG07) is purchased from Gelest Inc., PA, USA. The FITC-dextran (500 kMW, 3 kMw), 6 nm CdSeS/ZnS quantum dots, FITC-BSA protein (66 kDa), Rhodamine 6G, and the 30–40 nm large, 5–7 atomic layer thick water-dissolvable graphene nanoplatelets (799092) are purchased from Sigma Aldrich Inc., MO, USA. The 100 and 600 nm SiO_2_ particles coated with gold nanoparticles are synthetized in the lab (Supplementary Note 1). The fluorescence labeled urinary exosomes (M1075) and FITC-collagen (M1304, type-I, 300 nm in length and 1.6 nm in diameter) are purchased from BioVision Inc., CA, USA in the format of cryo-dried powders. Note that the exosomes are suspended in a low-conductivity isosmotic buffer (mainly 9.5% sucrose solution).

### Software and electronics

The device is powered with a sinusoidal AC signal from a function generator (DG 3102C, Tektronix Inc., PA, USA) and an amplifier (25A250A, Amplifier Research, PA, USA). The electric signals are measured with an oscilloscope (MSOX 2024A, Keysight Inc., CA, USA). The finite element simulations are performed on COMSOL and MATLAB. The fluorescence background for 50 and 28 nm scenarios are subtracted to reveal the patterns. The particle image velocimetry analysis is performed by Visual Studio C++ (Microsoft) and MATLAB Inc., USA following the calculation in Supplementary Note 2.

### Device fabrication and operations

In total, 5 nm Cr/200 nm Au are deposited on *Y*-41 lithium niobate wafer after standard photolithography. The SU-8 mold is fabricated on silicon wafer with a channel height of 25 μm or 10 μm. The channel window over the IDTs are 50 μm high to avoid collapse of the PDMS. The AENT devices have a thin ceiling for observing single nanoparticles. To reduce the background signals from nanoparticle adsorption, the device is modified with a thin ceiling (<500 μm) made of hard PDMS (Supplementary Fig. 4). The IDTs are bonded to wires with silver epoxy. The wavelengths for most AENT devices mentioned in this paper is 120 μm, except for Fig. 2a (80 μm), Fig. 3d Collagen (300 μm) and Fig. 4k, l (120 and 300 μm, harmonic interaction).

### Characterizing piezoelectric materials

The detailed procedures and discussion on the characterization of acoustoelectronic efficiency and acoustic streaming speeds of different piezoelectric materials are enclosed in Supplementary Figs. 5 and 6 and Supplementary Note 3 and 4. The corresponding abbreviations for different crystal are shown in Supplementary Table 1. The frequency-dependent data of surface acoustoelectronic efficiency and acoustic streaming speed are enclosed in Supplementary Fig. 7. Electric distortion may be a concern for surface potential measurements; however, the distortion will not impact the choice of the crystals, which is discussed in Supplementary Note 5 and Supplementary Fig. 8.

### Raman spectroscopy

The 633 nm HeNe laser is focused through a ×10 objective on the Rhodamine 6G aqueous sample (10^−4^ M). The spectra are collected using Jobin Yvon LabRam ARAMIS Horiba Inc., JP.

### Numerical simulation

The detailed procedures and discussions for simulations are enclosed in Supplementary Note 6, 7 and Supplementary Fig. 9. The parameter lists used for electric and acoustic simulation are attached in Supplementary Tables 2 and 3.

## Supplementary information

Supplementary Information

Description of Additional Supplementary Files

Supplementary Movie 1

Supplementary Movie 2

Supplementary Movie 3

Supplementary Movie 4

Supplementary Movie 5

Supplementary Movie 6

Supplementary Movie 7

## Data Availability

The data supporting the findings in this article are available from the corresponding author upon request. Source data are provided with this paper.

## Source data

Source Data

## Code availability

The codes related to computing acoustoelectronic contrasts are available at the Zenodo repository: https://zenodo.org/record/4547604#.YC3glmhKiUk.
