# Supplementary material for: Acoustoelectronic nanotweezers enable dynamic and large-scale control of nanomaterials
Source: Nat Commun. 2021 Jun 22;12:3844. doi: 10.1038/s41467-021-24101-z (PMC8219664; doi:10.1038/s41467-021-24101-z)
Supplement: Supplementary file 1 — Supplementary Information [file 41467_2021_24101_MOESM1_ESM.pdf]

Supplementary Information

Acoustoelectronic nanotweezers enable dynamic and large-scale control of nanomaterials

Peiran Zhang[1,*], Joseph Rufo[1,*], Chuyi Chen[1], Jianping Xia[1], Zhenhua Tian[1], Liying Zhang[1], Nanjing Hao[1], Zhanwei Zhong[2], Yuyang Gu[1], Krishnendu Chakrabarty[2], and Tony Jun Huang[1✉]

[1] Department of Mechanical Engineering and Materials Science, Duke University, NC 27708, USA

[2] Department of Electrical and Computer Engineering, Duke University, NC 27708, USA

[*] These authors contribute equally to this paper.

✉ To whom correspondence should be addressed. Email: tony.huang@duke.edu

Supplementary Figures

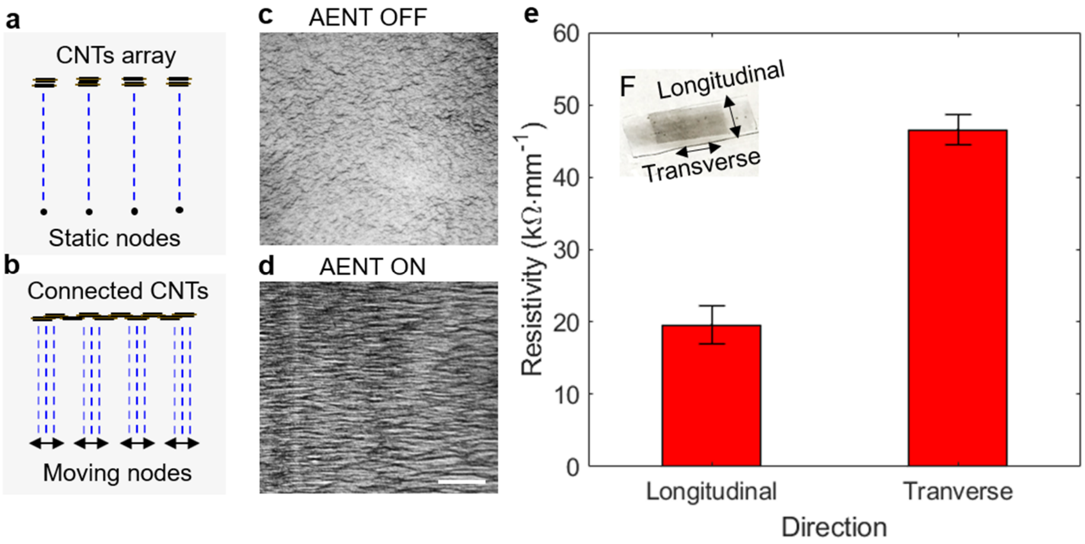

Supplementary Figure 1. Fabricating PDMS film with interconnected carbon nanotubes (CNTs) and associated anisotropic resistivities. (**a**) Schematic configuration for patterning CNTs gratings using static-nodes of AENT. (**b**) Schematic configuration for patterning interconnected CNTs using moving-nodes of AENT. (**c**) Microscopic image of PDMS film containing CNTs transferred from no wave region (*i.e.*, AENT OFF). (**d**) Microscopic image of PDMS film containing CNTs transferred from standing wave region (*i.e.*, AENT ON). (**e**) Anisotropic resistivities of the surface of PDMS film (*i.e.*, inset) containing interconnected CNTs. Scale bar: 60 µm.

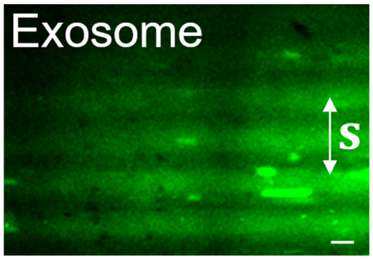

Supplementary Figure 2. 1D pattern of FITC-exosomes in isosmotic buffer using low amplitude excitation. The label '**s**' with bi-arrows the propagation direction of the acoustoelectronic waves. Scale bar: 30 µm.

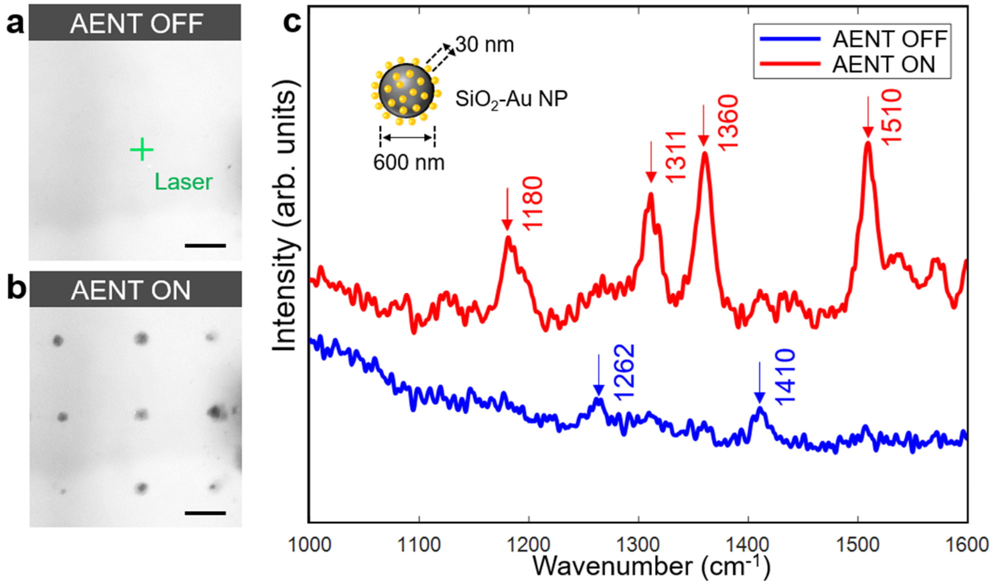

Supplementary Figure 3. Concentration of SERS probes. (**a**) The SERS probes are randomly dispersed in the sample solution (*i.e.*, 10^{-4} M Rhodamine 6G) when the AENT is off. The green cross indicates the focal position of 633 nm laser. (**b**) 2D patterning of SERS probes when AENT is on. (**c**) Raman spectra obtained at the laser focus when the AENT is OFF (*i.e.*, blue line) and ON (*i.e.*, red line). The SERS probes are 600 nm diameter SiO_2 particles surface coated with 30 nm diameter gold SERS beads that can be patterned using low excitation amplitudes. The blue and red arrows indicate the signature peaks of PDMS and Rhodamine 6G, respectively. Scale bars: 30 µm.

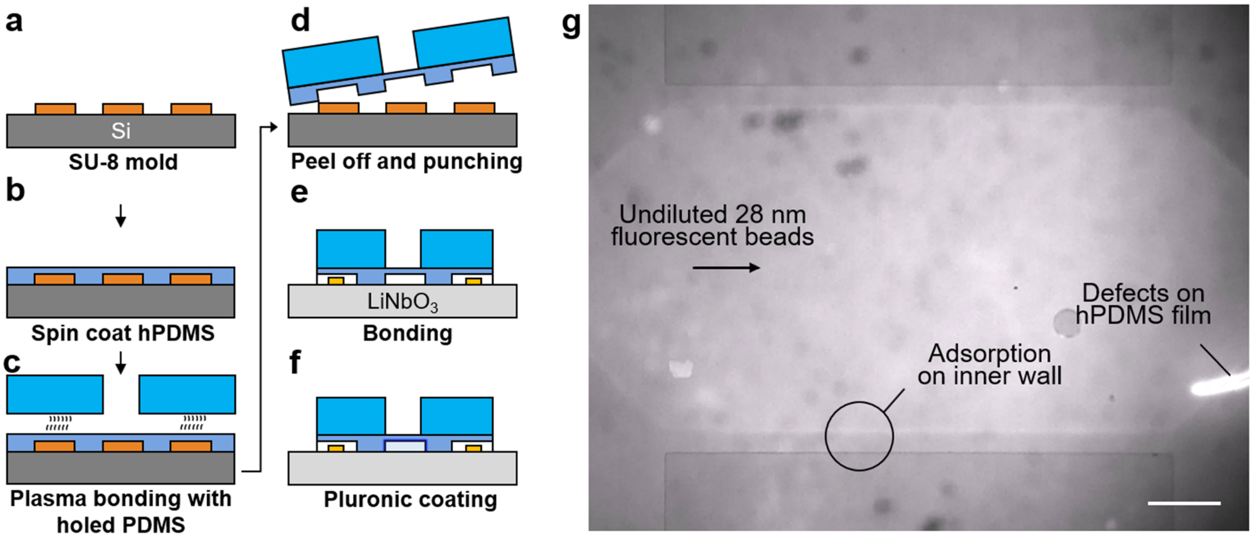

Supplementary Figure 4. Device fabrication. (**a**) – (**f**) Schematic pipeline for fabricating AENT devices with thin ceilings and hPDMS walls. (**g**) The reduced surface adsorption and diffusion into the wall coated with hPDMS (black circle) after 5 times of flushing of fluorescence 28 nm PS beads at maximum concentration. The defects on the hPDMS walls (*i.e.*, cracks) serve as a control that demonstrating the diffusion of small nano-beads into normal PDMS walls. Scale bar: 400 µm.

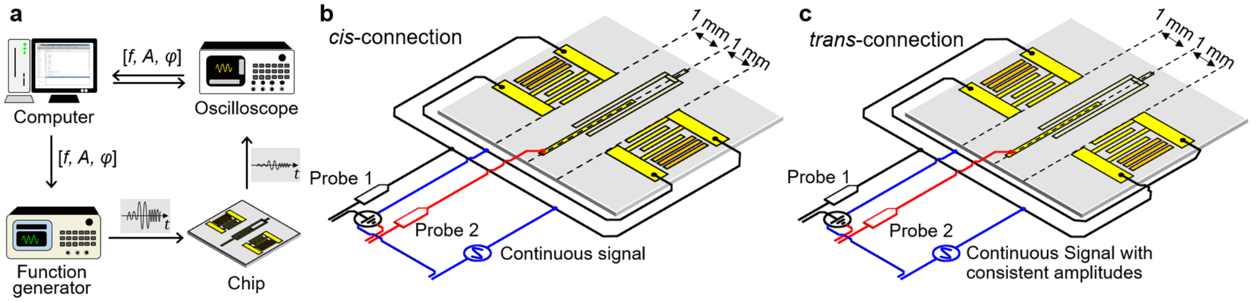

Supplementary Figure 5. Schematic setups for measuring the surface acoustoelectronic efficiency (κ_{AET}) for different piezoelectric substrates. (**a**) Measurement system setup. (**b**) Device configuration for measuring the amplitude of the AC surface electric potential of DAs (Φ_{cis}) using *cis*-electric connection of energizing the IDT pairs. (**c**) Device configuration for measuring the amplitude of the AC surface electric potential of DNs (Φ_{trans}) using *trans*-electric connection of energizing the IDT pairs. The measured excitation voltages are forcibly tuned to be consistent for *cis* and *trans* scenarios. Image credit for **Supplementary Figure 5a**: Peiran Zhang.

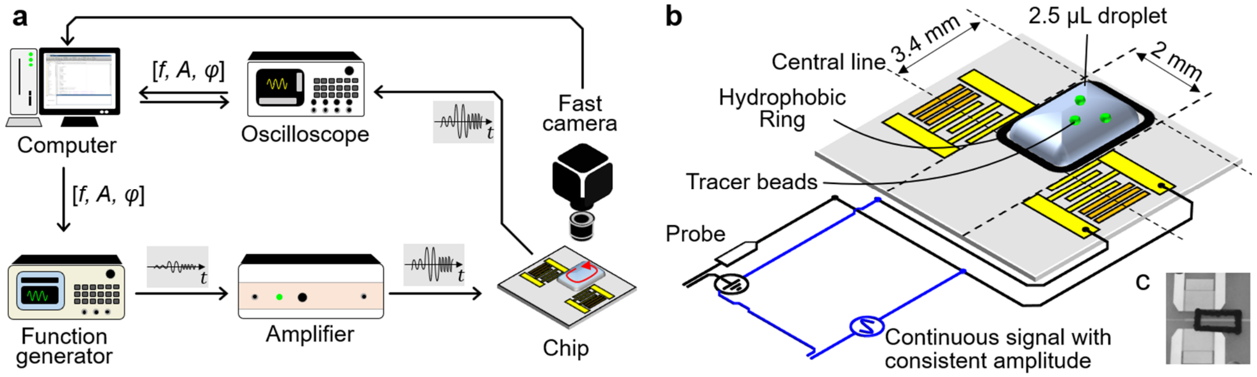

Supplementary Figure 6. Schematic setups for measuring the magnitude of time-averaged acoustic streaming speed (u_{fluid}). (**a**) Measurement system setup. (**b**) Device configuration for measuring the streaming speed in a rotating droplet with fluorescence tracer particles. The excitation voltages for measuring different piezoelectric materials are forcibly tuned to 5 Vpp. The black contour indicates the position of hydrophobic ring that confines the boundary of the droplet. (**c**) Device image with hydrophobic ring drew by marker pen. Image credit for **Supplementary Figure 6a**: Peiran Zhang.

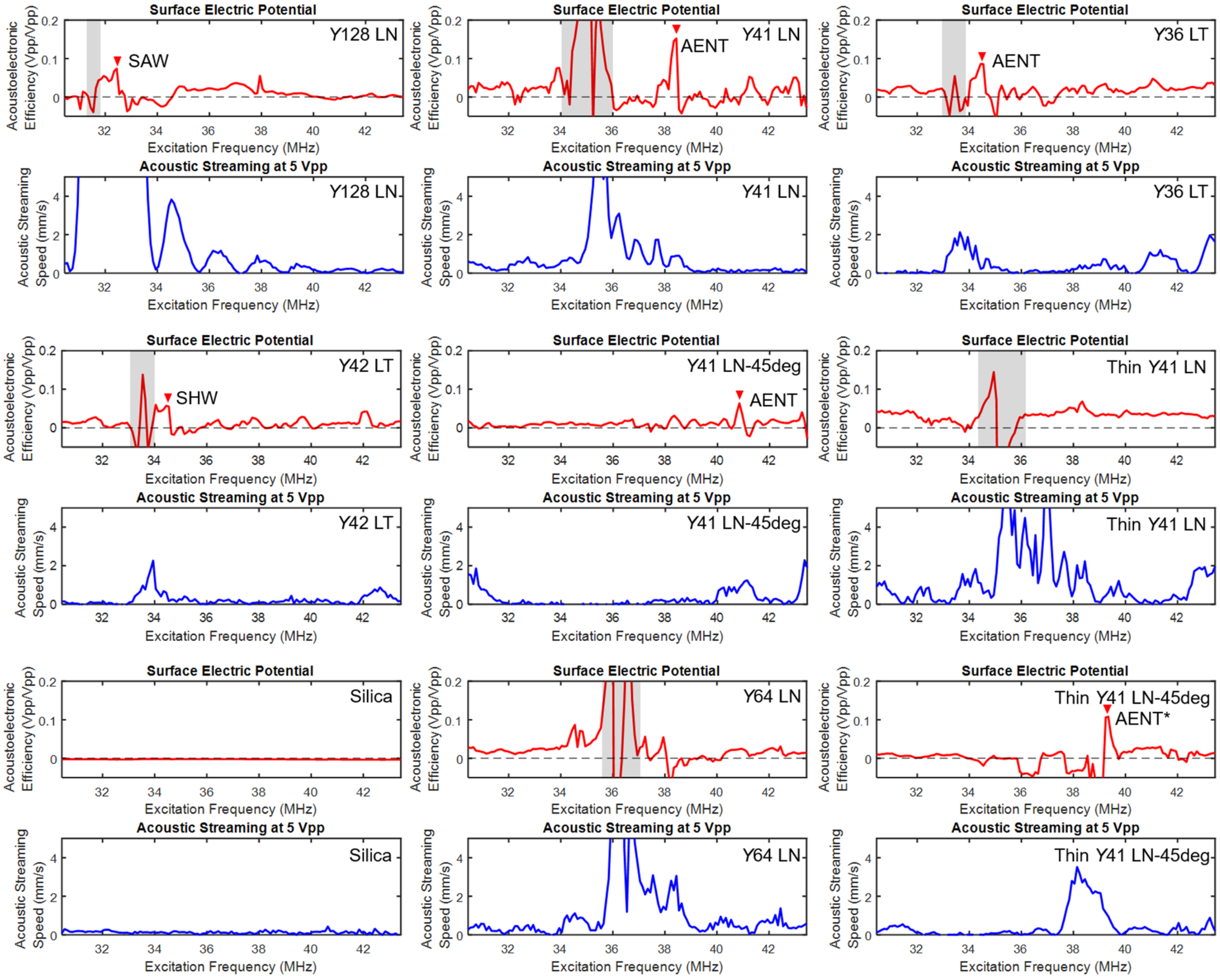

Supplementary Figure 7. Co-plotted data of frequency-dependent responses of surface acoustoelectronic efficiency (red lines) and frequency-dependent acoustic streaming speed (blue lines) for 9 piezoelectric substrates (**Supplementary Table 1**). The grey shadings indicate abnormal and non-consistent response region, which are excluded for generating the chart of **Fig. 1d**. The 'AENT' labels indicate the frequency used for AENT substrates. 'SHW' indicates the frequency used for generating shear-horizontal waves with previously published substrate *Y*-42 $LiTaO_3$. 'SAW' indicates the frequency used for generating surface acoustic waves on *Y*-128 $LiNbO_3$. The silica substrate does not have piezoelectricity and serves as a negative control.

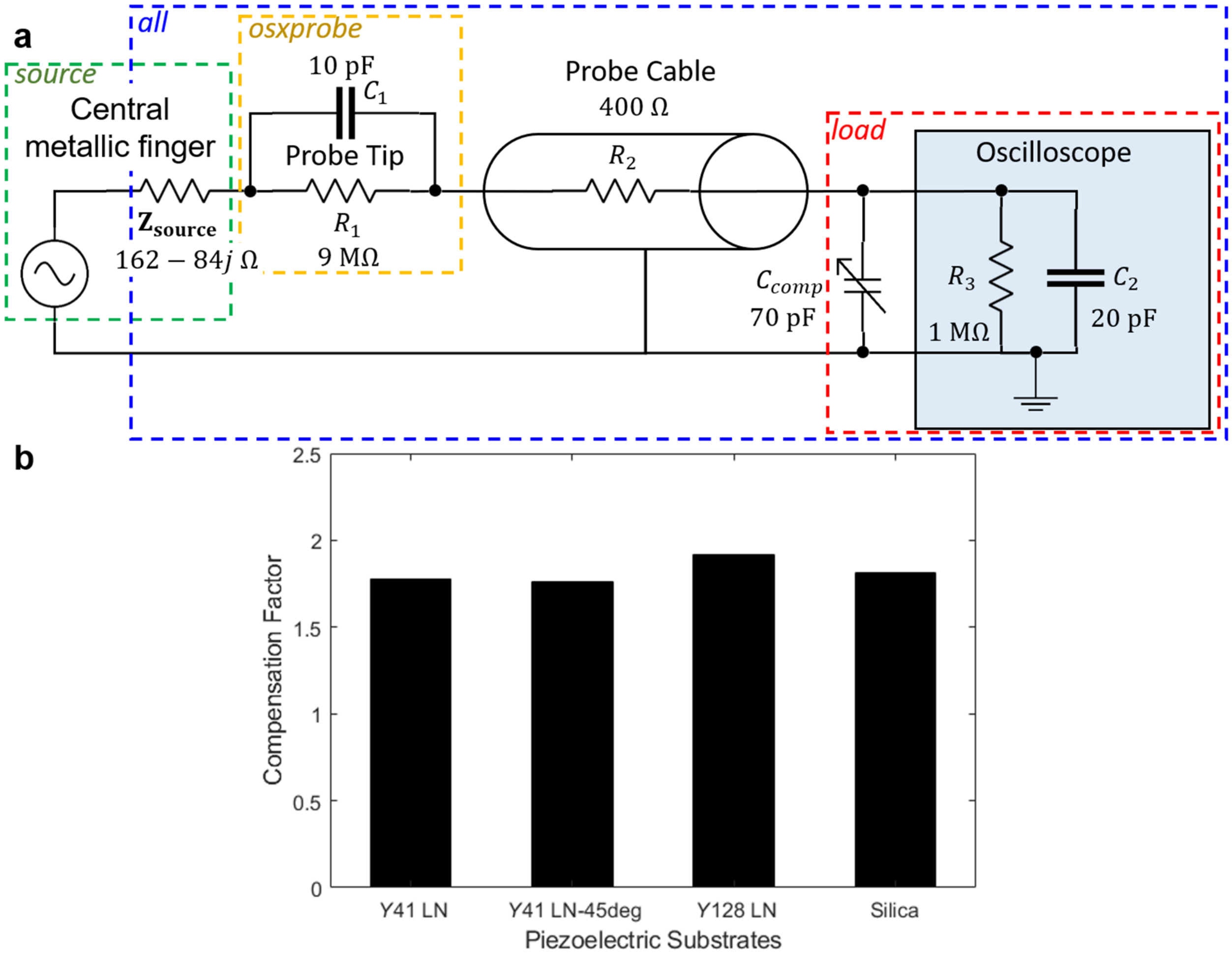

Supplementary Figure 8. Electric modelling of the probe for measuring the electric surface potential on different crystals. (**a**) Schematic electric model for the measurement system. The labels '*load*', '*osxprobe*', '*source*', '*all*', and the corresponding dashed boxes indicate corresponding models of oscilloscope, oscilloscope probe, central metallic finger, and the whole system, respectively. (**b**) The compensation factors for deriving the actual surface electric potential on different piezoelectric crystals.

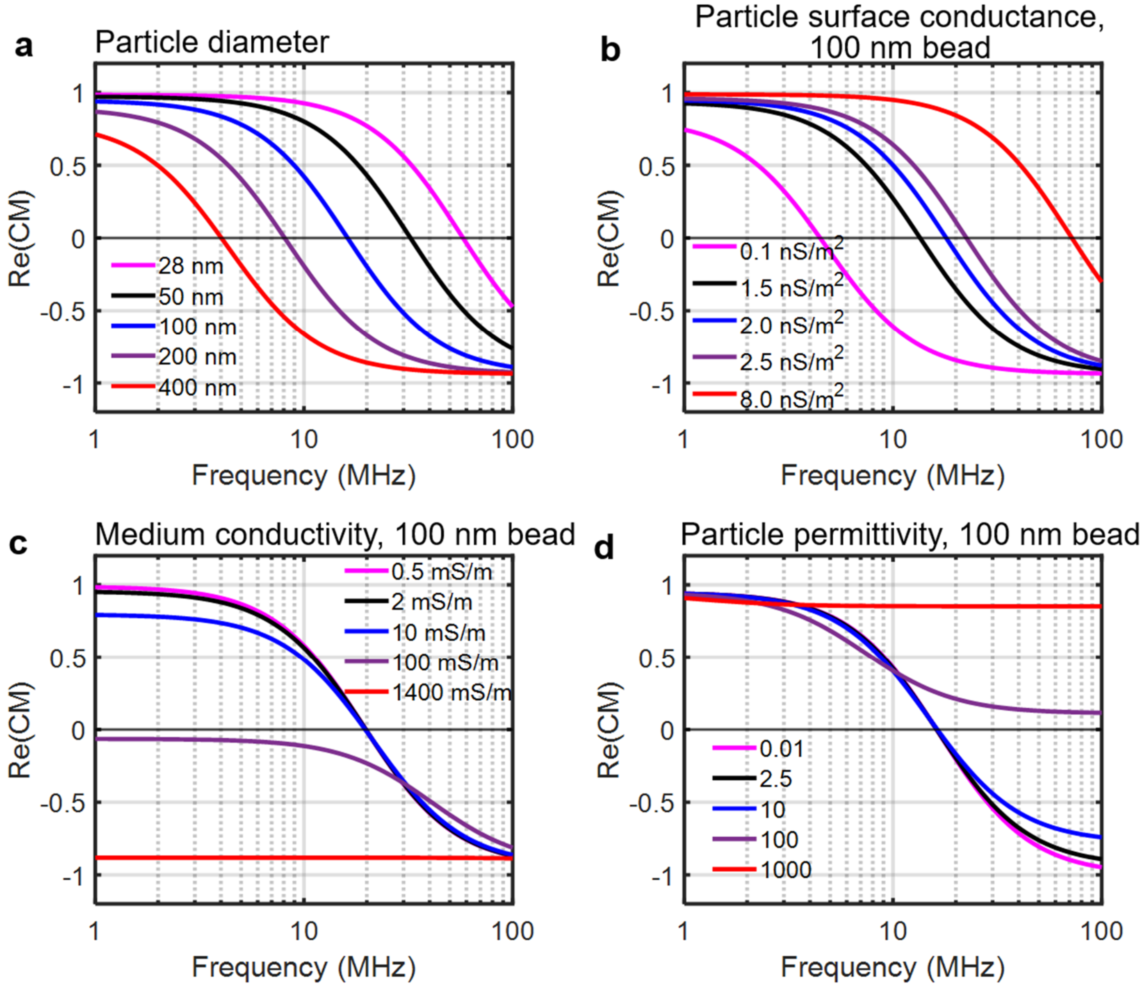

Supplementary Figure 9. Theoretical simulations of the frequency-dependent real-part of the CM factors of nanobeads with varied particle diameter (**a**), surface conductance (**b**), medium conductivity (**c**), and particle permittivity (**d**). For high-conductivity medium, the electric field will be shield by the thin fluid-layer near the substrate and cannot actuate the nanoparticles.

Supplementary Tables

Supplementary Table 1. Piezoelectric material candidates for AENT.

Name	Piezoelectric material	Wave direction	Notes
***Y*128 LN**	*Y*-128° $LiNbO_3$, DSP[†], 0.5 mm	*X*-axis of crystal	Used for SAW[§]
***Y*41 LN**	*Y*-41° $LiNbO_3$, SSP[‡], 0.5 mm	*X*-axis of crystal	Used in AENT[‖]
***Y*36 LT**	*Y*-36° $LiTaO_3$, SSP, 0.5 mm	*X*-axis of crystal	Used in AENT
***Y*42 LT**	*Y*-42° $LiTaO_3$, SSP, 0.5 mm	*X*-axis of crystal	Used for SHW[#]
***Y*41 LN-45deg**	*Y*-41° $LiNbO_3$, SSP, 0.5 mm	45°-rotated *X*-axis of crystal	
**Thin *Y*41 LN**	*Y*-41° $LiNbO_3$, SSP, 0.18 mm	*X*-axis of crystal	
**Silica**	Silica wafer, DSP, 0.5 mm	N/A	Negative control
***Y*64 LN**	*Y*-64° $LiNbO_3$, SSP, 0.5 mm	*X*-axis of crystal	Can be used for AENT
**Thin *Y*41 LN-45deg**	*Y*-41° $LiNbO_3$, SSP, 0.18 mm	45°-rotated *X*-axis of crystal	Can be used for AENT

Notes: [†] DSP: double side polished. [‡] SSP: single side polished. [§] SAW: surface acoustic wave. [‖] AENT: acoustoelectronic nanotweezers. [#] SHW: shear-horizontal wave.

Supplementary Table 2. Parameters list for calculating CM factors of nanoparticles and vesicles.

Parameter	Description	Value
f	Acoustic frequency	1 – 100 MHz
ω	Angular frequency	2πf
r	Particle radius	50 nm
σ_m	Conductivity of medium	2×10^{-3} S·m^{-1}
σ_p	Conductivity of particle	10^{-14} S·m^{-1}
σ_s	Surface conductivity of particle	1.8×10^{-9} S·m^{-2}
ε_m	Dielectric constant of medium	$80\varepsilon_0$
ε_p	Dielectric constant of particle	$2.55\varepsilon_0$
ε_0	Dielectric constant of vacuum	8.85×10^{-12} F·m^{-1}
σ_{mem}	Conductivity of membrane of single-shell particle	2.5×10^{-7} S·m^{-1}
σ_{plasma}	Conductivity of inner space of single-shell particle	2×10^{-1} S·m^{-1}
σ_{mem_s}	Surface conductivity of membrane of single-shell particle	10×10^{-9} S·m^{-2}
ε_{mem}	Dielectric constant of membrane of single-shell particle	$3\varepsilon_0$
ε_{plasma}	Dielectric constant of inner space of single-shell particle	$75\varepsilon_0$
R	Inner radius of single-shell particle	44×10^{-9} m
d	Membrane thickness of single-shell particle	6×10^{-9} m

Supplementary Table 3. Acoustic parameters for the finite element simulation of AENT.

Parameter	Description	Value
h	Channel height	10, 25 μm
λ	Wavelength of acoustic waves	120 μm
r	Particle radius	50 nm
c_p	Longitudinal wave speed of polystyrene nanoparticle	1950 $m \cdot s^{-1}$
ρ_p	Density of polystyrene nanoparticle	1060 $kg \cdot m^{-3}$
c_m	Sound speed of water	1495 $m \cdot s^{-1}$
ρ_m	Density of water	997 $kg \cdot m^{-3}$
c_s	Sound speed of used wave mode on Y41 $LiNbO_3$ substrate	4612.8 $m \cdot s^{-1}$
ρ_s	Density of Y41 $LiNbO_3$	4650 $kg \cdot m^{-3}$
c_{PDMS}	Longitudinal wave speed of PDMS	1080 $m \cdot s^{-1}$
ρ_{PDMS}	Density of PDMS	970 $kg \cdot m^{-3}$
μ	Dynamic viscosity of water (25 °C)	1×10^{-3} Pa·s

Supplementary Notes

Supplementary Note 1. Synthesizing nanoparticles for SERS detection

Synthesis of Au nanoparticles (Au NPs). Au NPs with the size of ~30 nm were prepared according to previously reported method. [1] 2.5 mL of chloroauric acid (0.1 M) was added into 100 mL of ultrapure water and then heated to boiling under magnetic stirring. After quickly injecting 1.5 mL of trisodium citrate (10 %, w/w), the mixed solution was refluxed for ~30 min until the color of solution became wine red. The resultant solution gradually cooled to room temperature under stirring and was stored in refrigerator at 4 °C for further use.

Synthesis of SiO_2 nanoparticles (SiO_2 NPs). Uniform spherical silica particles with the sizes of ~100 nm and ~600 nm were prepared by seeded growth based on the Stöber method. [2]

For the synthesis of ~100 nm SiO_2, 300 μL of tetramethylethylenediamine and 3 mL of H_2O were first mixed with 35 mL of ethanol under vigorous stirring. Then 2.25 mL of tetraethyl orthosilicate (TEOS) was added into above solution. After this addition, the whole mixture was allowed to react for 12 h, and the as-prepared SiO_2 dispersions were washed with ethanol by centrifugation (14,000 rpm, 10 min) three times. Finally, the SiO_2 nanoparticles powder was obtained after drying at 60 °C for 12 h.

For the synthesis of ~600 nm SiO_2, a hydrolysis solution was first obtained by mixing 2 mL of ammonium hydroxide, 20 mL of ethanol, and 10 mL of H_2O. Then, 3 mL of the hydrolysis solution and 0.5 mL of TEOS were added into 50 mL three-necked flasks under an ice bath and rapidly stirred for 10 min to form the seed solution. During the growth process, 12 mL of hydrolysis solution and 12 mL of TEOS/ethanol solution (the ratio of TEOS to ethanol is 3:5 by volume) were simultaneously dropped into the seed solution through different necks at a rate of 100 μL/min. After this addition, the whole mixture was allowed to react for 12 h, and the as-prepared SiO_2 dispersions were washed with ethanol by centrifugation (5,000 rpm, 15 min) three times. Finally, the SiO_2 nanoparticles powder was obtained after drying at 60 °C for 12 h.

Synthesis of Au-coated silica nanoparticles (SiO_2-Au NPs). Au NPs were coated on the surface of SiO_2 by chemical modification. To obtain SiO_2-Au NPs, amino-functionalized SiO_2 (SiO_2-NH_2) was first prepared through adding 200 μL of 3-aminopropyltriethoxysilane (APTS) to 10 ml of SiO_2-ethanol solution (3 mg/mL). After reacting for 8 h, the mixture was centrifuged (14,000 rpm, 5 min) and washed with ethanol three times before being dispersed into 4 mL of ultrapure water.

Then, 87 μL of 3-mercaptobenzoic acid, 20 mg of EDC and 2 mL of SiO_2-NH_2 solution were added to 2 mL of ultrapure water, and the mixed solution was shaken for 8 h at room temperature. To remove excess Au NPs and EDC, the above solution was centrifuged (10,000 rpm, 5 min) and washed with ultrapure water three times. Finally, the SiO_2-Au NPs were re-dispersed into 1 mL of ultrapure water for further use.

Supplementary Note 2. Calculation of the actuation forces on single 100 nm and 400 nm particles on acoustoelectronic nanotweezers

The force balances of a nanoparticle being manipulated by acoustoelectronic nanotweezers is governed by Supplementary Equation 1:

$$m_p\mathbf{a} = \mathbf{F} - 6\pi\eta r\mathbf{v} \quad (1)$$

m_p, $\mathbf{a}$, η, r, and $\mathbf{v}$ are the mass of the nanoparticle, the acceleration of particle, dynamic viscosity of medium, the radius of nanoparticles, and the moving speed of particle. $\mathbf{F}$ is considered composite force acting on those single nanoparticles, and the influence from the acousto-dielectrophoresis will be dominating as the radius of particle becomes larger. $\mathbf{a}$ and $\mathbf{v}$ are derived from the PIV analysis of time-serial fluorescence images.

Supplementary Note 3. Characterizing surface acoustoelectronic efficiency (κ_{AET}) of piezoelectric materials

The goal of this section is to measure the ratio (κ_{AET}) between the amplitude of received AC surface electric potentials (Φ) for different piezoelectric crystals with frequency dependency (*i.e.*, 30.44 MHz – 43.44 MHz).

One challenge for measuring the surface acoustoelectronic efficiencies is the interference of radio frequency (RF) signals either in air or between the testing instruments, leading to irregular electric responses over nearly the full frequency spectrum for certain crystals (*e.g.*, *Y*-41 $LiNbO_3$) and making the direct electric measurement of travelling pulse envelopes impossible. Thus, we opted to measure the surface electric potential (Φ) using a continuous standing wave configuration instead and acoustic streaming speed for different crystals, which is more suitable in the context of AENT. Specifically, the surface electric potential (Φ) is evaluated by calculating the differences between the measured amplitudes of continuous standing waves at the center-line of two opposing

IDTs with 0 and π phase-shifts (*e.g.*, nodes or antinodes for *trans* or *cis* connection), which greatly enhance the signal and eliminates received RF radiation background (**Supplementary Figure 5a**).

As shown in **Supplementary Figure 5b** and **5c**, we put a single-finger metallic probe (20 μm width) at the center-line of 2 opposing IDTs. These IDTs are separated by 2 mm and have carefully designed reflectors to enhance the resonance of acoustoelectronic waves. The concept underlying the method is to use the amplitude of received signal under *cis*- (**Supplementary Figure 5b**) connection of the IDT pairs to subtract that under *trans* (**Supplementary Figure 5c**) connection of the IDT pairs. The derived differences can be considered as the amplitude differences over DAs and DNs with 0 and π phase differences. Based on this concept, the backgrounds that obscure the AC surface potentials, including the radiation background from both the air and the substrate, and the noise originating from the irregular electric interactions or unknown sources, can be eliminated. Strictly speaking, we cannot claim that all background noise is completely removed, because the RF interactions between these instruments and the devices can be extremely complicated; however, this method can remove most of the noise effectively and has correctly reflected the potentials of different piezoelectric materials for the demonstration and validation of AENT in experiments. Note that our method cannot exactly resolve the modes of surface displacements (*i.e.*, *x*, *y*, or *z*, **Fig. 1a**). This is because all the modes of displacements may generate transient electric charges coupled on the local deformation. However, the out-of-plane vibrations (*i.e.*, *z*-mode vibrations) can be resolved by the PIV experiments discussed in **Supplementary Note 4**. For resolving the in-plane vibrations (*i.e.*, *x*- and *y*-mode vibrations), the signs of the κ_{AET} implies which mode is dominating the vibration, since the *y*-mode vibrations (parallel to wave propagation) will lead to a phase-shift of π and negative signs for subtracting the measured amplitude under *trans*-scenario (**Supplementary Figure 5c**) from that under *cis*-scenario (**Supplementary Figure 5b**).

Take '*Y*41 LN' for example, at the 'AENT' frequency, the chart shows a peak value of approximately +0.16 $Vpp \cdot Vpp^{-1}$, indicating the *x*-mode vibrations (0 phase-shift, perpendicular to wave-propagation) are dominating the displacement at 38.44 MHz. Another piezoelectric material, *Y*-42 $LiTaO_3$, known for generating shear-horizontal waves ('SHW', solid red diamond) also shows very low acoustic streaming speed at certain excitation frequencies, yet its κ_{AET} is ~ 60 % lower than that of *Y*-36 $LiTaO_3$. As a negative control of no piezoelectricity, a silica wafer is tested due to its centrosymmetrical crystallographic structure. The silica shows nearly zero surface

acoustoelectronic efficiency (κ_{AET}) and acoustic streaming speed (u_{fluid}). However, the zero κ_{AET} at certain excitation frequencies does not necessarily mean that there is no displacement on the surface, since the κ_{AET} is not uniform around the rotational orientations of the displacement , or the mixed vibrations of *x*-mode and *y*-mode displacements may cancel the surface charges out due to the inherent phase difference of π in standing wave fields.

Notably, the excitation voltages for *cis*- and *trans*- connections can vary even if when the same values are set on the function generator (*i.e.*, 6 Vpp). To make the AC potentials consistent in both scenarios, the measured excitation voltages for the applied frequency component under the *cis*-scenarios are firstly recorded for a given voltage setup on the function generator, and then the measured excitation voltages at the applied frequency component under *trans*-scenarios are forcibly tuned to be as the same as the recorded *cis*-scenarios through real-time feed-back controlling codes. Furthermore, with the co-grounding electric connection configurations shown in **Supplementary Figure 5b** and **5c**, no significant variations of the measured AC surface potentials are observed when the shapes of the wires are changed. The data are shown by the red lines in **Supplementary Figure 7**. Silica substrate does not have piezoelectricity and serve as a negative control. We also co-screened the excitation amplitudes and excitation frequencies, and it seems that the excitation amplitudes do not have significant effect on κ_{AET} when < 20 Vpp.

Supplementary Note 4. Measuring acoustic streaming speed (u_{fluid}) on different piezoelectric materials

To enable nano-manipulation with AENT, the out-of-plane vibrations and associated acoustics-induced hydrodynamic disturbances need to be minimized. To measure the acoustic energy leaked into fluid for different piezoelectric substrates, we performed measurements on the averaged acoustic streaming speed within a 2.5 μL water droplet using consistent measured excitation voltages for different frequencies and crystals. As shown in **Supplementary Figure 6**, the water droplet is placed in the hydrophobic ring tangential to the dashed 'central line'. Once the IDT is activated, the 10 μm fluorescence PS beads will be rotated with flow and the trajectories will be recorded. The excitation frequency will be automatically shifted throughout the range of interest (*i.e.*, 30.44 – 43.44 MHz) while recording the fluorescence videos using a fast camera. The measured excitation voltages are forcibly tuned to 5 Vpp *via* real-time feedback control. The

acquired videos are processed by homemade PIV codes, and the time-averaged particle speeds are plotted with respect to the excitation frequencies.

The previous attempts on acoustic pressure measurement using hydrophone have failed due to electric interferences. Although the magnitude of acoustic streaming does not linearly corelated to the amplitude of out-of-plane vibrations as acoustic pressure does, the streaming speed reflects the relative potentials of different piezoelectric materials for generating acousto-hydrodynamic disturbances.

Supplementary Note 5. Electronic modelling of the setup for surface electric potential measurement

The measured acoustoelectronic efficiencies for different piezoelectric crystals in **Fig. 1d** and **Supplementary Figure 7** have not been compensated from the perspective of high-frequency electronics for the measurement setup. In this note, we provide an electrical model on measurement compensation and deriving the actual surface electric potential amplitudes in a standing acoustoelectronic wave field.

The electrical model of the measurement setup is shown in **Supplementary Figure 8a**. For the electrical measurement scenarios discussed in **Supplementary Note 3**, the central metallic probe within the standing wave region can be considered as a power source of sinusoidal voltages. The induced voltages first go through the central metal probe with an impedance $\mathbf{Z_{source}}$ of $162-84j\ \Omega$ for $Y41$ $LiNbO_3$. Note that this impedance is measured at the frequency of 38.44 MHz using the impedance measurement instrument (Keysight E5063A). Next, this voltage signal is picked up by a 10:1 oscilloscope passive probe (Keysight N2842A), which can be modeled as the parallel connection of a $10\ pF$ capacitor (C_1) and a $9\ M\Omega$ resistor (R_1). The voltage signals go through a lossy coaxial cable with a resistance of $400\ \Omega$ (R_2) and finally are picked up by the oscilloscope. The internal load of the oscilloscope can be modeled as the parallel connection of a 20 pF capacitor (C_2) and a $1\ M\Omega$ resistor (R_3). Between the probe cable and the oscilloscope, a tunable capacitor (C_{comp}) is used to calibrate waveform deformation, and the capacitance should satisfy: $\frac{R_1}{R_3}=\frac{C_2+C_{comp}}{C_1}$. Therefore, it can be calculated that $C_{comp}=70\ pF$ was used in the measurement setup.

Assuming the overall impedance is Z_{all} and the impedance of the oscilloscope is $\mathbf{Z_{load}}$, the voltage measured by the oscilloscope can be expressed using the follow equations (Supplementary Equation 2 - 9):

$$|\mathbf{Z_{load}}| = \frac{1}{\sqrt{(\frac{1}{R_3})^2 + (2\pi f(C_2 + C_{comp}))^2}} \quad (2)$$

$$\emptyset_{load} = tan^{-1}(-2\pi f(C_2 + C_{comp})R_3) \quad (3)$$

$$\mathbf{Z_{load}} = |\mathbf{Z_{load}}|\ cos\emptyset_{load} + |\mathbf{Z_{load}}|\ sin\emptyset_{load} \cdot j \quad (4)$$

$$|\mathbf{Z_{osxprobe}}| = \frac{1}{\sqrt{(\frac{1}{R_1})^2 + (2\pi f C_1)^2}} \quad (5)$$

$$\emptyset_{osxprobe} = tan^{-1}(-2\pi f C_1 R_1) \quad (6)$$

$$\mathbf{Z_{osxprobe}} = \left|\mathbf{Z_{osxprobe}}\right|\ cos\emptyset_{osxprobe} + \left|\mathbf{Z_{osxprobe}}\right|\ sin\emptyset_{osxprobe} \cdot j \quad (7)$$

$$\mathbf{Z_{all}} = \mathbf{Z_{source}} + \mathbf{Z_{osxprobe}} + R_2 + \mathbf{Z_{load}} \quad (8)$$

$$V_{load} = \frac{V_{source}}{|\mathbf{Z_{all}}|}\ |\mathbf{Z_{load}}| \cdot 10 = 0.59 \cdot V_{source} \quad (9)$$

where the symbols with labels '*load*', '*osxprobe*', '*source*', and '*all*' indicate the parameters (*i.e.*, impedance **Z**, phase ∅) associated with oscilloscope, oscilloscope probe, central metallic finger, and the whole system, respectively (**Supplementary Figure 8a**). Based on the above calculation, the voltage shown in the oscilloscope is only 59% of that actually applied on the wire. Note that different substrates have different electric characteristics at 38.44 MHz, therefore the actual calibration factors vary depending on the materials and rotation angles of the IDT (**Supplementary Figure 8b**). The calculated surface potentials need to be further compensated by multiplying by 1.047 to obtain the correct peak-peak potential of the tested standing wave field, due to the geometric averaging effect of a 20 μm width metal probe aligned with the antinodes of the acoustoelectronic waves with a sinusoidal periodicity of 60 μm. Thus, the compensation factor for the actual surface electric potential on *Y*41 $LiNbO_3$ is 1.047×1/0.59 = 1.78.

Supplementary Note 6. Prediction of nanoparticle behaviors in alternating current electric fields created by acoustoelectronic nanotweezers

The time-average force acting on the nanoparticle is given by Supplementary Equation 10:

$$\langle F_{AE} \rangle = 2\pi r^3 \varepsilon_m \mathrm{Re}(\mathbf{CM}) \nabla \left| \vec{\mathbf{E}}_{\mathbf{rms}} \right|^2 \tag{10}$$

r, ε_m, $\mathbf{CM}$, and $\vec{\mathbf{E}}_{\mathbf{rms}}$ are the radius of nanoparticle, the dielectric constant of medium, the Clausius-Mossotti (CM) factor of nanoparticle, and the RMS value of electric field strength.

The CM factor of solid, homogeneous nanoparticles in the alternating current electric field can be predicted by the calculation below (Supplementary Equation 11 – 14):

$$\mathbf{CM}_{\sigma} = \frac{\boldsymbol{\sigma}_{\mathbf{c}} - \boldsymbol{\sigma}_{\mathbf{m}}}{\boldsymbol{\sigma}_{\mathbf{c}} + \boldsymbol{\sigma}_{\mathbf{m}}} \tag{11}$$

$$\boldsymbol{\sigma}_{\mathbf{m}} = \sigma_m - i\omega\varepsilon_m \,;\ \boldsymbol{\sigma}_{\mathbf{c}} = \sigma_c - i\omega\varepsilon_p \,;\ \sigma_c = \sigma_p + \frac{2\sigma_s}{r} \tag{12}$$

$$\mathbf{CM}_{\boldsymbol{\varepsilon}} = \frac{\boldsymbol{\varepsilon}_{\mathbf{p}} - \boldsymbol{\varepsilon}_{\mathbf{m}}}{\boldsymbol{\varepsilon}_{\mathbf{p}} + \boldsymbol{\varepsilon}_{\mathbf{m}}} \tag{13}$$

$$\boldsymbol{\varepsilon}_{\mathbf{m}} = \varepsilon_m + i\frac{\sigma_m}{\omega};\ \boldsymbol{\varepsilon}_{\mathbf{p}} = \varepsilon_p + i\frac{\sigma_c}{\omega} \tag{14}$$

$\mathbf{CM}_{\sigma}$ and $\mathbf{CM}_{\boldsymbol{\varepsilon}}$ are the CM factor calculated from the conductivity and permittivity, respectively. σ_m, σ_p, ε_m, ε_p, ω, and r are the conductivities of the medium and the particle, the dielectric constants of the medium and the particle, the angular frequency of the acoustic waves, the radius of the particle, respectively. σ_c and σ_s are the composite conductivity and surface conductivity of the particle. The values of related parameters are shown in Supplementary Table 2.

The CM factor of spherical, vesicle-like nanoparticles in the alternating current electric field can be predicted by the single-shell model below (Supplementary Equation 15 - 19):

$$\mathbf{CM}_{\mathbf{v}} = \frac{\boldsymbol{\varepsilon}_{\mathbf{v}} - \boldsymbol{\varepsilon}_{\mathbf{m}}}{\boldsymbol{\varepsilon}_{\mathbf{v}} + 2\boldsymbol{\varepsilon}_{\mathbf{m}}} \tag{15}$$

$$\boldsymbol{\varepsilon}_{\mathbf{m}} = \varepsilon_m + i\frac{\sigma_m}{\omega};\ \boldsymbol{\varepsilon}_{\mathbf{v}} = \frac{(\gamma^3 + 2\mathbf{K}_{\mathbf{23}})}{\gamma^3 - \mathbf{K}_{\mathbf{23}}} \tag{16}$$

$$\mathbf{K}_{\mathbf{23}} = \frac{\boldsymbol{\varepsilon}_{\mathbf{plasma}} - \boldsymbol{\varepsilon}_{\mathbf{mem}}}{\boldsymbol{\varepsilon}_{\mathbf{plasma}} + 2\boldsymbol{\varepsilon}_{\mathbf{mem}}};\ \gamma = \frac{R + d}{R} \tag{17}$$

$$\boldsymbol{\varepsilon}_{\mathbf{mem}} = \varepsilon_{mem} + i\frac{\sigma_{mem\_c}}{\omega};\ \boldsymbol{\varepsilon}_{\mathbf{plasma}} = \varepsilon_{plasma} + i\frac{\sigma_{plasma}}{\omega} \tag{18}$$

$$\sigma_{mem\_c} = \sigma_{mem} + \frac{2\sigma_{mem\_s}}{r} \tag{19}$$

$\mathbf{CM}_{\mathbf{v}}$ is the CM factor of single shell particle. σ_m, σ_{mem}, σ_{plasma}, ε_m, ε_{mem}, ε_{plasma}, ω, and r are the conductivities of the medium, the vesicle membrane, and the vesicle plasma, the dielectric constants of the medium , the vesicle membrane, and the vesicle plasma, the angular frequency of the acoustic waves, the radius of the particle, respectively. σ_{mem_c} and σ_{mem_s} are the composite

conductivity and surface conductivity of the vesicle membrane. R and d are the inner radius of the vesicle and the thickness of the membrane, respectively. The values of related parameters are shown in Supplementary Table 2. The $\mathbf{CM}_{\mathrm{v}}$ and the behavior of vesicle-like nanoparticle are sensitive to surface conductivity, which is sensitive to the zeta-potential and the conductance of membrane.

Supplementary Note 7. Numerical simulation of AENT

In this work, the mechanical vibration and the electric potential in the substrate, as well as the electric potential and acoustic field in the liquid were numerically simulated for analyzing the acoustoelectronic force (AEF) and acoustic radiation force (ARF) in AENT. The mechanical vibration and the electrical response in the 41° Y-X $LiNbO_3$ substrate are governed by the following equations (Supplementary Equation 20 - 21):

$$T_{ij} = C_{ijkl}^{E} \cdot S_{kl} - e_{ijk}^{T} \cdot E_k \tag{20}$$

$$D_i = e_{ikl} \cdot S_{kl} + \epsilon_{ij}^{S} \cdot E_k \tag{21}$$

where the stress vector (T_{ij}), the strain vector (S_{kl}), the electrical displacement (D_i), and the electric field (E_k) are coupled; and the C_{ijkl}^{E}, e_{ijk}, and ϵ_{ij} stand for the elasticity matrix, the piezoelectric matrix, and the permittivity matrix, respectively. In the liquid, electrostatic field is governed by the following equations (Supplementary Equation 22 - 23):

$$\mathbf{E} = -\nabla V \tag{22}$$

$$\nabla \cdot (\varepsilon_0 \varepsilon_r \mathbf{E}) = \rho_v \tag{23}$$

where $\mathbf{E}$ is the electric field, V is the electric potential, ε_0 is the permittivity of the vacuum, ε_r is the relative permittivity of the liquid, and ρ_v is a space charge density. The harmonic acoustic field is governed by the mass and momentum conservation equations (Supplementary Equation 24 - 25):

$$j\omega\rho + \rho_0 \nabla \cdot (\mathbf{v}) = 0 \tag{24}$$

$$\rho_0 j\omega \mathbf{v} = -c_0^2 \nabla\rho + \mu\nabla^2\mathbf{v} + \left(\frac{1}{3}\mu + \mu_B\right)\nabla(\nabla \cdot \mathbf{v}) \tag{25}$$

where ρ is the density perturbation, $\mathbf{v}$ is the medium vibration velocity, and ω is the angular frequency of the acoustic field; ρ_0, c_0, μ, and μ_B are the stasis density, speed of sound, dynamic viscosity, and bulk viscosity of the medium, respectively; and j is the imaginary unit ($j^2 = -1$).

The constitutive relation between acoustic pressure (p) and density perturbation (ρ) can be written as the follow equation (Supplementary Equation 26):

$$p = c_0^2 \rho \tag{26}$$

Based on these governing equations, COMSOL Multiphysics Version 5.4 (the COMSOL group) was used to simulate the physics coupled between the substrate and the loading liquid. The predefined "Solid Mechanic" interface was applied to the substrate for the solution of the mechanical vibration. The "Electrostatics" interface was applied to both of the substrate and the liquid fields to solve the electrical behavior in these fields. And the "Thermoviscous Acoustic" was applied to solve the acoustic field in the liquid. The "Piezoelectric Effect" Multiphysics condition was applied to the boundary between the two fields to the substrate field to couple the mechanical vibration and the electrical behavior in the substrate. And, the "Thermoviscous Acoustic-Structure Boundary", which constrains the velocity continuity, was applied to the boundary between the substrate and liquid. The positive and negative electric potential was applied to the IDT fingers alternatively as the activation of the device. Additionally, the mentioned interfaces were solved together either in frequency domain at the resonance frequency for the time-averaged solution of the substrate displacement, electrical field, and acoustic field or using a time dependent solver for the dynamic behavior of these fields.

Based on the solution of acoustic pressure (p) and medium vibration velocity ($\mathbf{v}$)time-averaged acoustic radiation force work on the nano particles can be calculated by the classic Gorkov's model (Supplementary Equation 27 - 29):

$$F_{rad} = -\nabla \left\{ V_p \left[\frac{f_1}{2\rho_0 c_0^2} \langle p^2 \rangle - \frac{3\rho_0 f_2}{4} \langle \mathbf{v} \cdot \mathbf{v} \rangle \right] \right\} \tag{27}$$

$$f_1 = 1 - \frac{\rho_0 c_0^2}{\rho_p c_p^2} \tag{28}$$

$$f_2 = \frac{2(\rho_p - \rho_0)}{2\rho_p + \rho_0} \tag{29}$$

where V_p is the volume of the particle, and ρ_p and c_p are its density and speed of sound velocity. Using Gorkov's model, the calculated acoustic radiation forces are ~20 times smaller than the AEF for a 100 nm particle. However, for nano-scale manipulation, the thermoviscous boundary effects will significantly enhance the acoustic contrast factors of nanoparticles, increasing the acoustic radiation forces to the same magnitude of AEF in the updated model (*i.e.*, the "small-

width boundary layers" regime, *Phys. Rev. E*, 92, 043010, 2015), but the former is still no larger than AEF. Despite the discrepancies between the two models, the dominance of acoustoelectronic effects is validated by patterning 100 nm particles over half-shielded substrates as shown in Fig. 2a.

For simulating the 2D AENT with orthogonal wave interferences, the displacement of SH waves in both directions can be written as the follow equations (Supplementary Equation 30 - 31):

$$\mathbf{u_1} = U_1[e^{i(\omega_1 t - k_{1x}x - k_{1x}y)} + e^{i(\omega_1 t + k_{1x}x + k_{1y}y + \varphi_1)}] \quad (30)$$

$$\mathbf{u_2} = U_2[e^{i(\omega_2 t - k_{2x}x + k_{2y}y)} + e^{i(\omega_2 t + k_{2x}x - k_{2y}y + \varphi_2)}] \quad (31)$$

Here, U_1 and U_2 are the displacement amplitude of both directions; φ_1 and φ_2 are the phase difference of the two IDTs in both directions, respectively. When the frequencies f_1 and f_2 of both directions are the same, the angular frequency $\omega_1=\omega_2=2\pi f_1$, and the components of wave number $k_{1x} = k_{1y} = k_{2x} = k_{2y} = \sqrt{2}/(2\lambda)$, where λ is the wavelength of acoustic waves. The voltage distribution and DEP force is calculated by coupling the Electrostatics and Solid Mechanic modules in COMSOL Multiphysics®. When the frequencies f_1 and f_2 are different, the voltage distributions V_1 and V_2 for both directions are first calculated, respectively. Then the total voltage distribution V changing with time t is calculated by $V = |V_1|sin(\omega_1 t) + |V_2|sin(\omega_2 t)$ using Algebraic Equation module in COMSOL Multiphysics®. The time averaging operator is calculated in the range of $[0,1/|f_2-f_1|]$.

Supplementary References

[1]. Zhang, L., Jin, Y., Mao, H., Zheng, L., Zhao, J., Peng, Y., Du, S., and Zhang, Z., Structure-selective hot-spot Raman enhancement for direct identification and detection of trace penicilloic acid allergen in penicillin. *Biosens. Bioelectron*. **2014**, *58*, 165-171.

[2]. Stöber, W., Fink, A., and Bohn, E., Controlled Growth of Monodisperse Silica Spheres in the Micron Size Range. *J. Colloid Interface Sci.* **1968**, *26*, 62-69.
