# Supplementary material for: Acoustoelectronic nanotweezers enable dynamic and large-scale control of nanomaterials
Source: Nat Commun. 2021 Jun 22;12:3844. doi: 10.1038/s41467-021-24101-z (PMC8219664; doi:10.1038/s41467-021-24101-z)
Supplement: Supplementary file 2 — Description of Additional Supplementary Files [file 41467_2021_24101_MOESM2_ESM.pdf]

Description of Additional Supplementary Files

Supplementary Movie 1. Patterning of 100 nm PS nanobeads on half-shield substrate (λ = 80 μm, (1/2)) and 110 nm PS beads (λ = 120 μm, (2/2)) with streaming using 1D AENT. Scale bars: 60 μm.

Supplementary Movie 2. Patterning of 50 nm (1/2) and 28 nm (2/2) PS nanobeads using 1D AENT. Scale bars: 240 μm.

Supplementary Movie 3. Abnormal patterns of fluorescent exosomes in isosmotic buffer (1/2) and 100 nm PS nanobeads in water (2/2) under high-amplitude excitation signals. Scale bars: 60 μm.

Supplementary Movie 4. Transient displacement distribution of pulsed travelling acoustoelectronic waves forming time-of-flight (ToF) AENT.

Supplementary Movie 5. Harmonic merging and splitting of 100 nm PS beads using two frequency components (λ_1 = 120 μm, λ_2 = 300 μm). Scale bar: 60 μm.

Supplementary Movie 6. Dynamic manipulation of CNTs. Continuously growing CNT pattern in flow with 1D AENT (1/3). Pattern transformation of CNTs using 2D AENT (2/3). Scale bars: 60 μm. On-demand orientation control of CNTs with 2D AENT (3/3). Scale bar: 15 μm.

Supplementary Movie 7. Single nano-particle manipulation. (1/2) Patterning 400 nm PS beads and (2/2) repeated pairing and release of single 400 nm particles using 2D AENT. Scale bars: 50 μm.
